# Supplementary figures and images for: A nascent polypeptide sequence modulates DnaA translation elongation in response to nutrient availability
Source: eLife. 2021 Sep 15;10:e71611. doi: 10.7554/eLife.71611 (PMC8443254; doi:10.7554/eLife.71611)

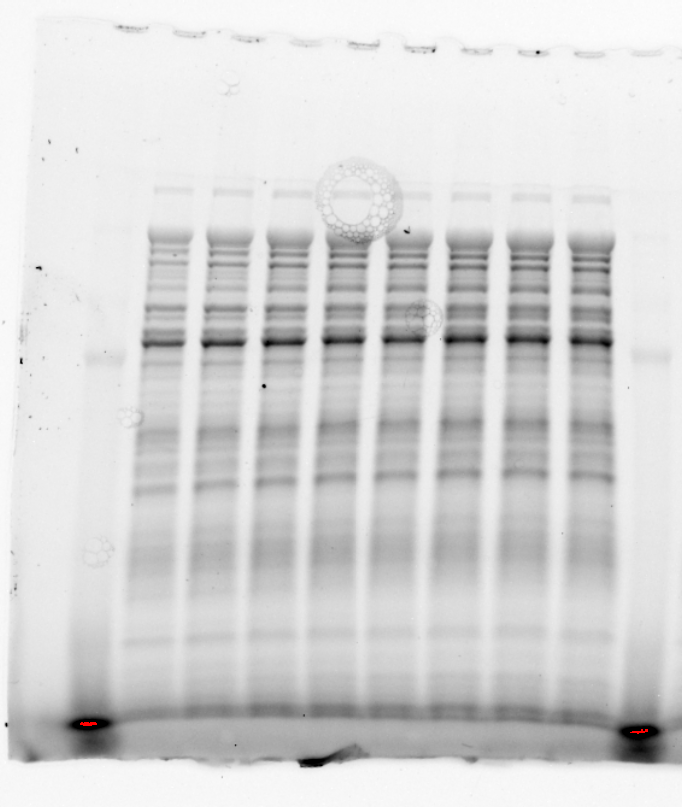

Supplement: Figure 1—source data 1. [file elife-71611-fig1-data1.zip › Figure 1-source data 1/panel A/Replicate2/PAGE_rep2.tif]

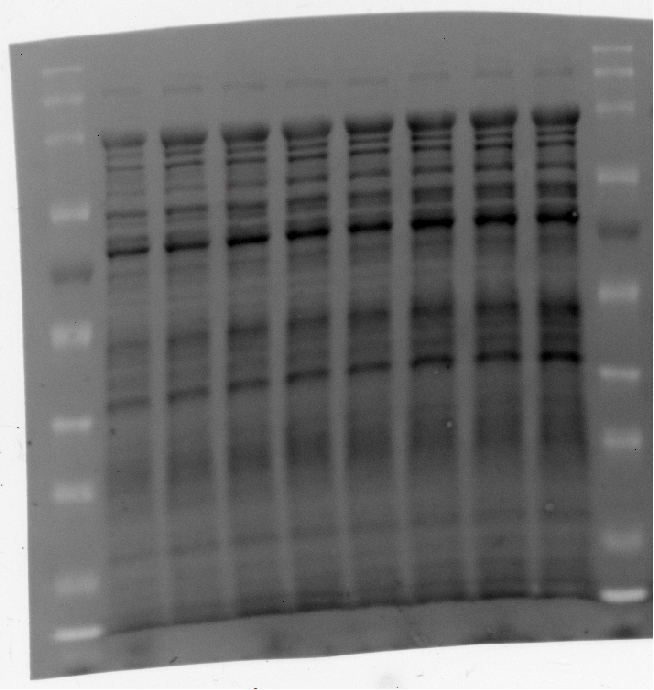

Supplement: Figure 1—source data 1. [file elife-71611-fig1-data1.zip › Figure 1-source data 1/panel A/Replicate2/transfer_rep2.tif]

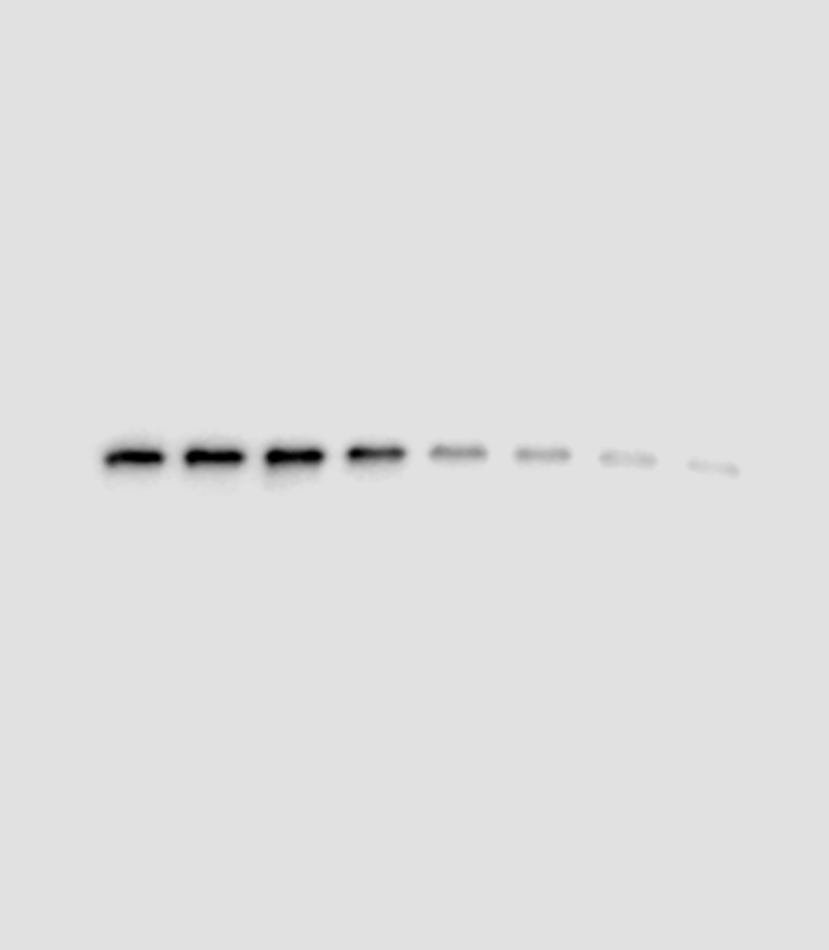

Supplement: Figure 1—source data 1. [file elife-71611-fig1-data1.zip › Figure 1-source data 1/panel A/Replicate2/WB_rep2_DnaA.tif]

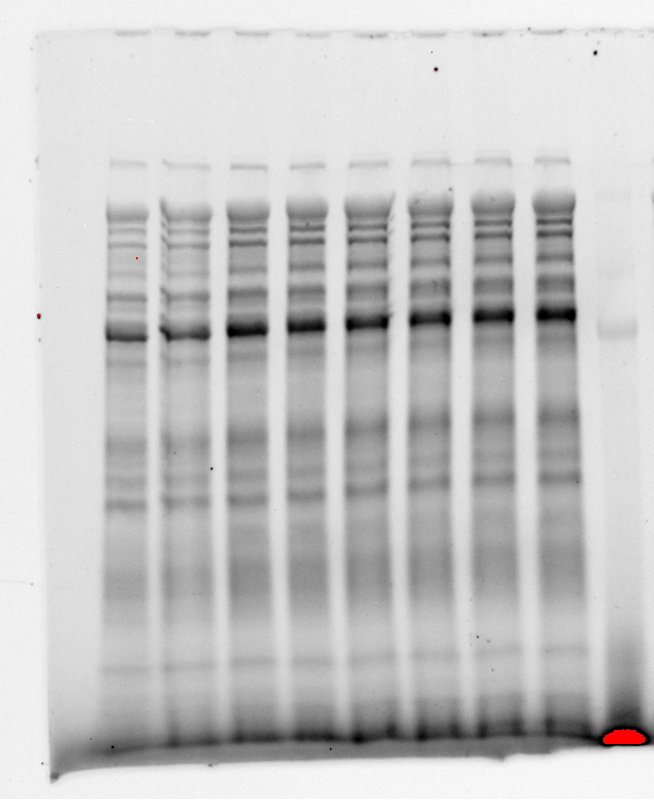

Supplement: Figure 1—source data 1. [file elife-71611-fig1-data1.zip › Figure 1-source data 1/panel A/Replicate3/PAGE_rep3.tif]

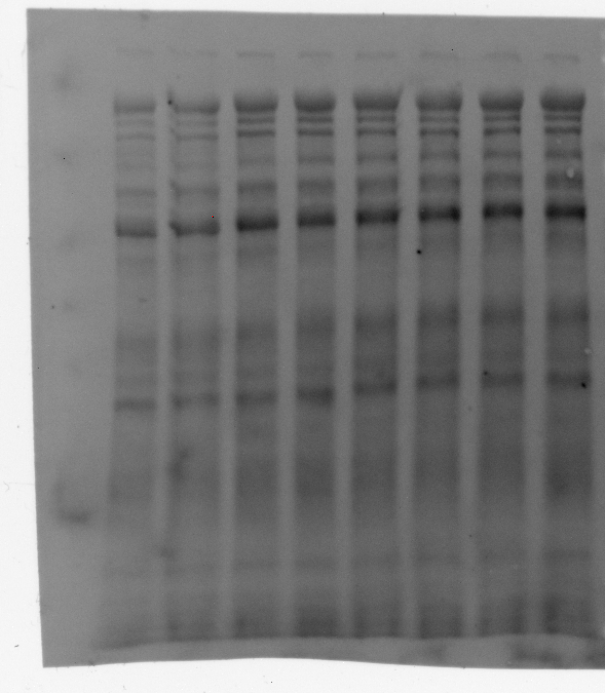

Supplement: Figure 1—source data 1. [file elife-71611-fig1-data1.zip › Figure 1-source data 1/panel A/Replicate3/transfer_rep3.tif]

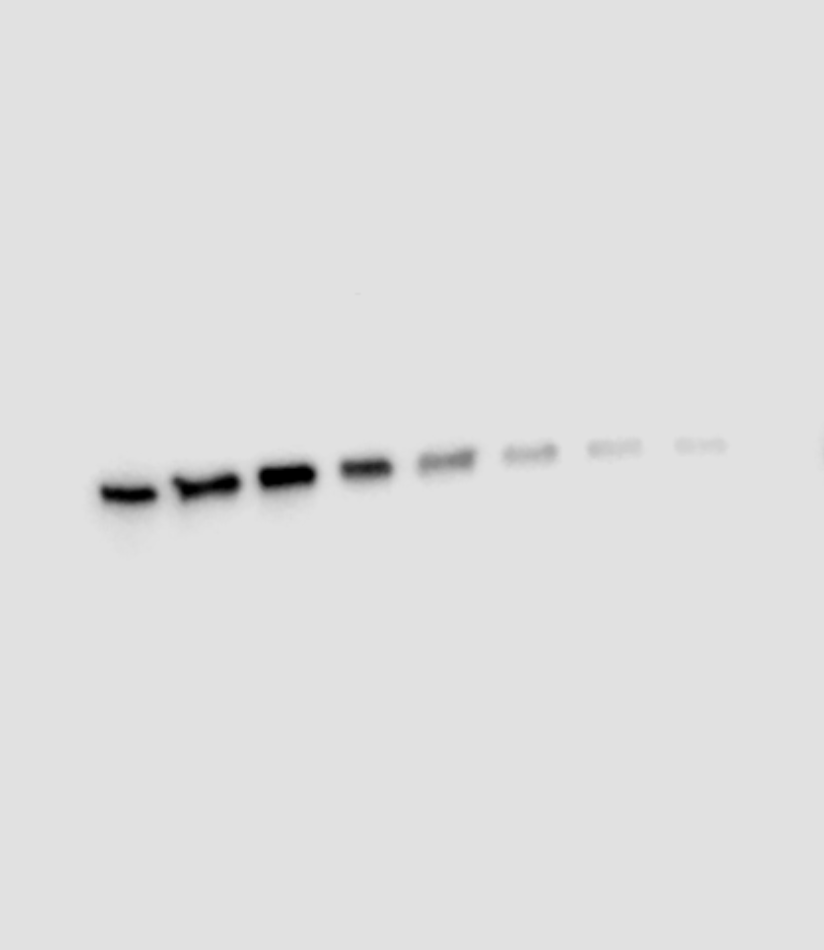

Supplement: Figure 1—source data 1. [file elife-71611-fig1-data1.zip › Figure 1-source data 1/panel A/Replicate3/WB_rep3_DnaA.tif]

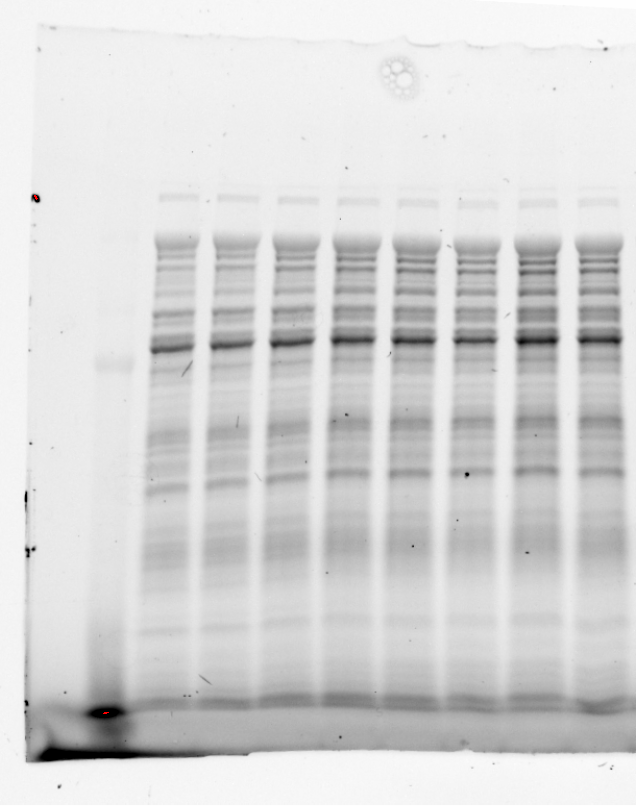

Supplement: Figure 1—source data 1. [file elife-71611-fig1-data1.zip › Figure 1-source data 1/panel A/Replicate1/PAGE_rep1.tif]

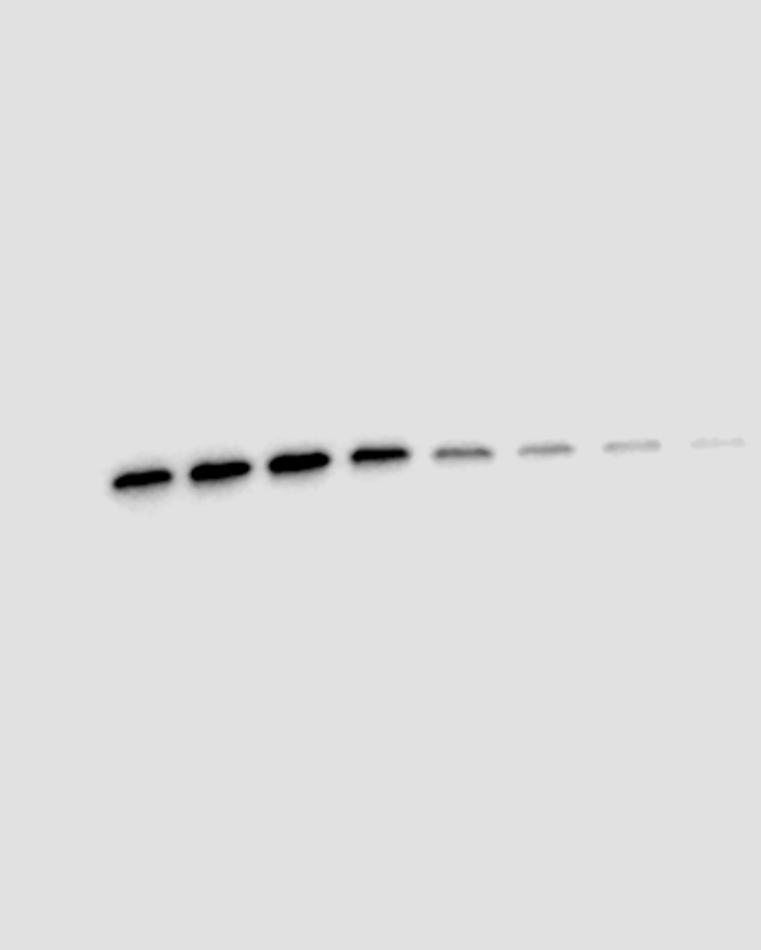

Supplement: Figure 1—source data 1. [file elife-71611-fig1-data1.zip › Figure 1-source data 1/panel A/Replicate1/WB_rep1_DnaA.tif]

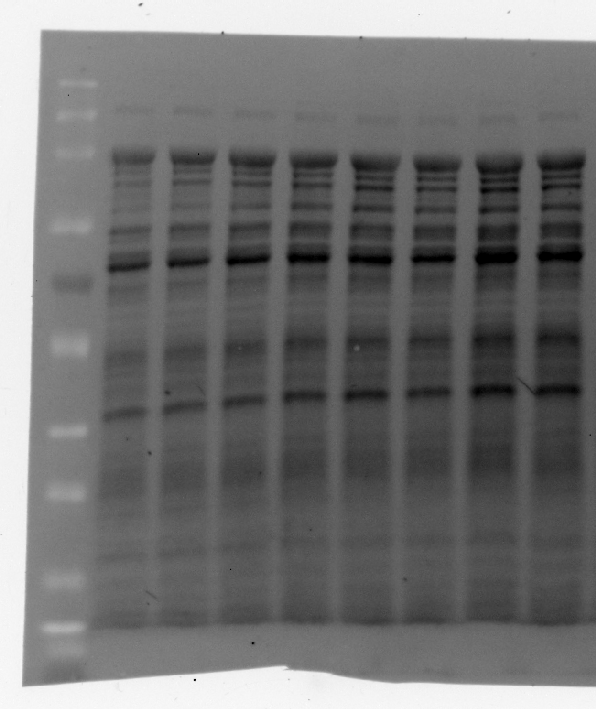

Supplement: Figure 1—source data 1. [file elife-71611-fig1-data1.zip › Figure 1-source data 1/panel A/Replicate1/Transfer_rep1.tif]

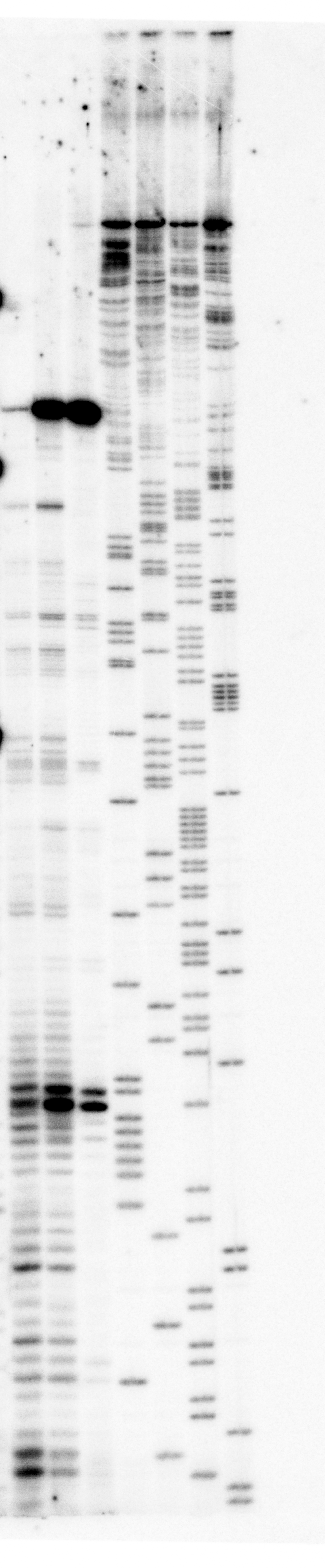

Supplement: Figure 1—figure supplement 1—source data 1. [file elife-71611-fig1-figsupp1-data1.zip › Figure 1-figure supplement 1-source data 1/panel D/RNaseV1_indirect_detection_PrimerB.tif]

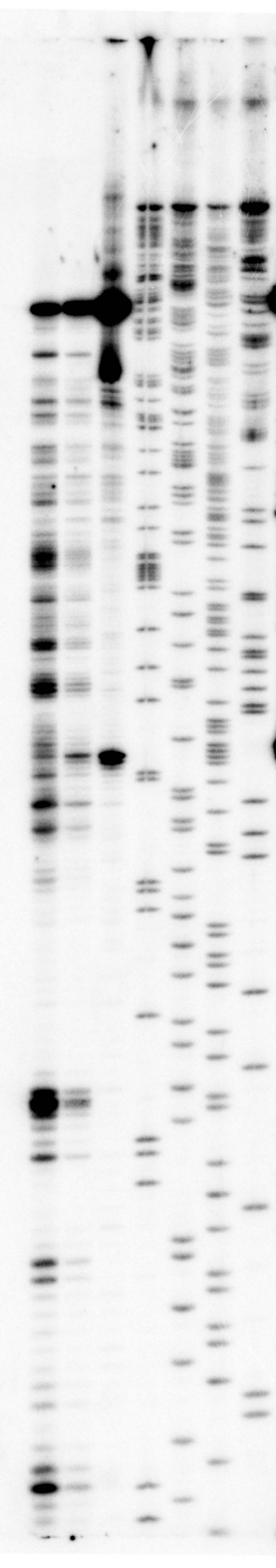

Supplement: Figure 1—figure supplement 1—source data 1. [file elife-71611-fig1-figsupp1-data1.zip › Figure 1-figure supplement 1-source data 1/panel D/RNaseV1_indirect_detection_PrimerA.tif]

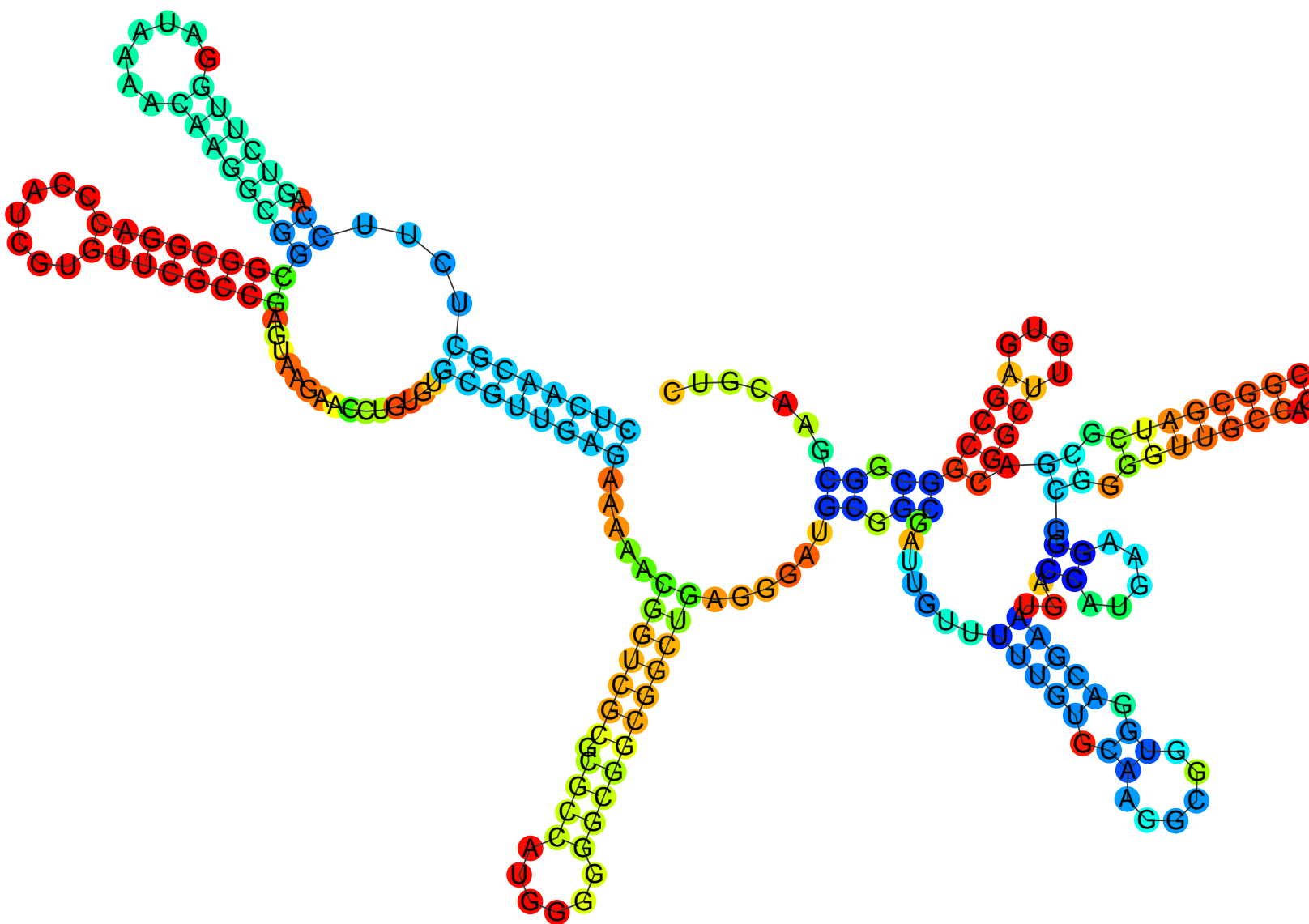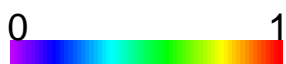

Supplement: Figure 1—figure supplement 1—source data 1. [file elife-71611-fig1-figsupp1-data1.zip › Figure 1-figure supplement 1-source data 1/mFold_ViennaRNA_predictions/ViennaRNA233.pdf]

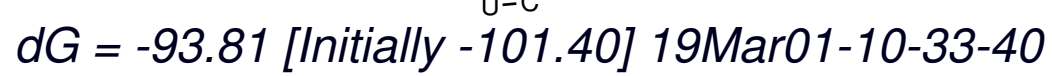

Supplement: Figure 1—figure supplement 1—source data 1. [file elife-71611-fig1-figsupp1-data1.zip › Figure 1-figure supplement 1-source data 1/mFold_ViennaRNA_predictions/19Mar01-10-33-40_253.pdf]

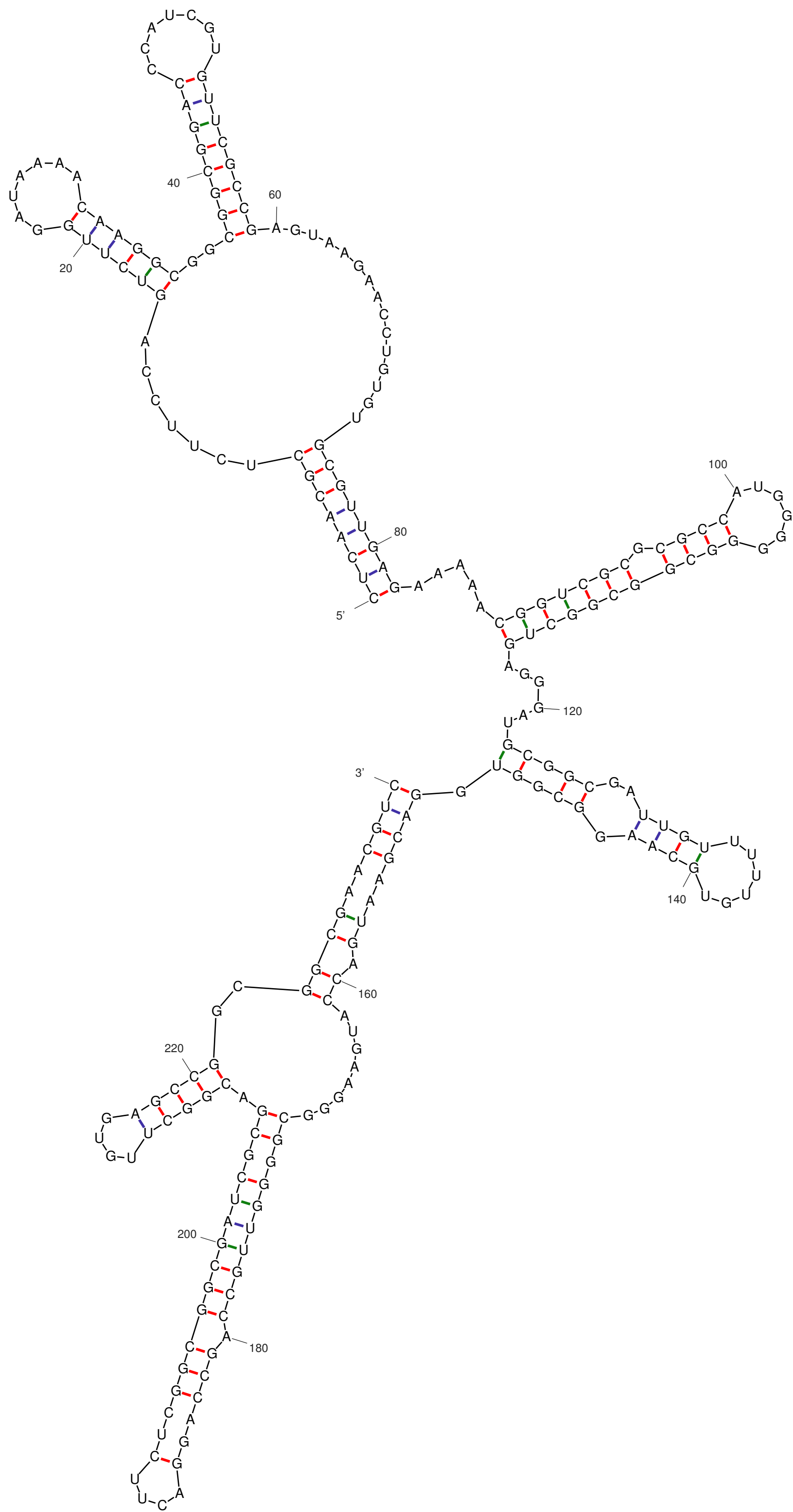

*dG = -89.11 [Initially -94.50] 19Mar01-10-27-50*

Supplement: Figure 1—figure supplement 1—source data 1. [file elife-71611-fig1-figsupp1-data1.zip › Figure 1-figure supplement 1-source data 1/mFold_ViennaRNA_predictions/19Mar01-10-27-50_233.pdf]

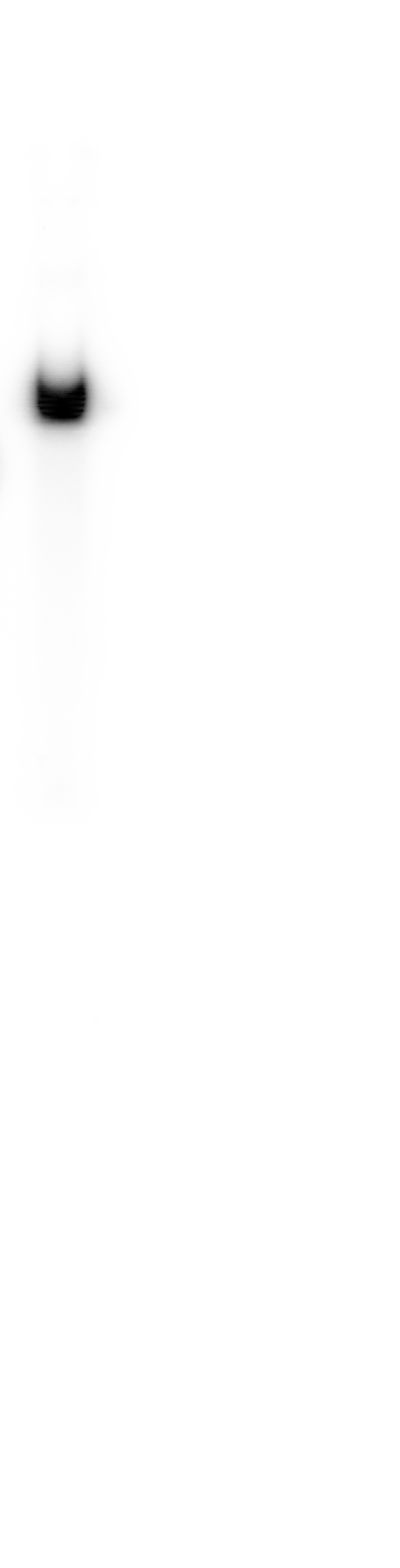

Supplement: Figure 1—figure supplement 1—source data 1. [file elife-71611-fig1-figsupp1-data1.zip › Figure 1-figure supplement 1-source data 1/panel A/Native_PAGE.tif]

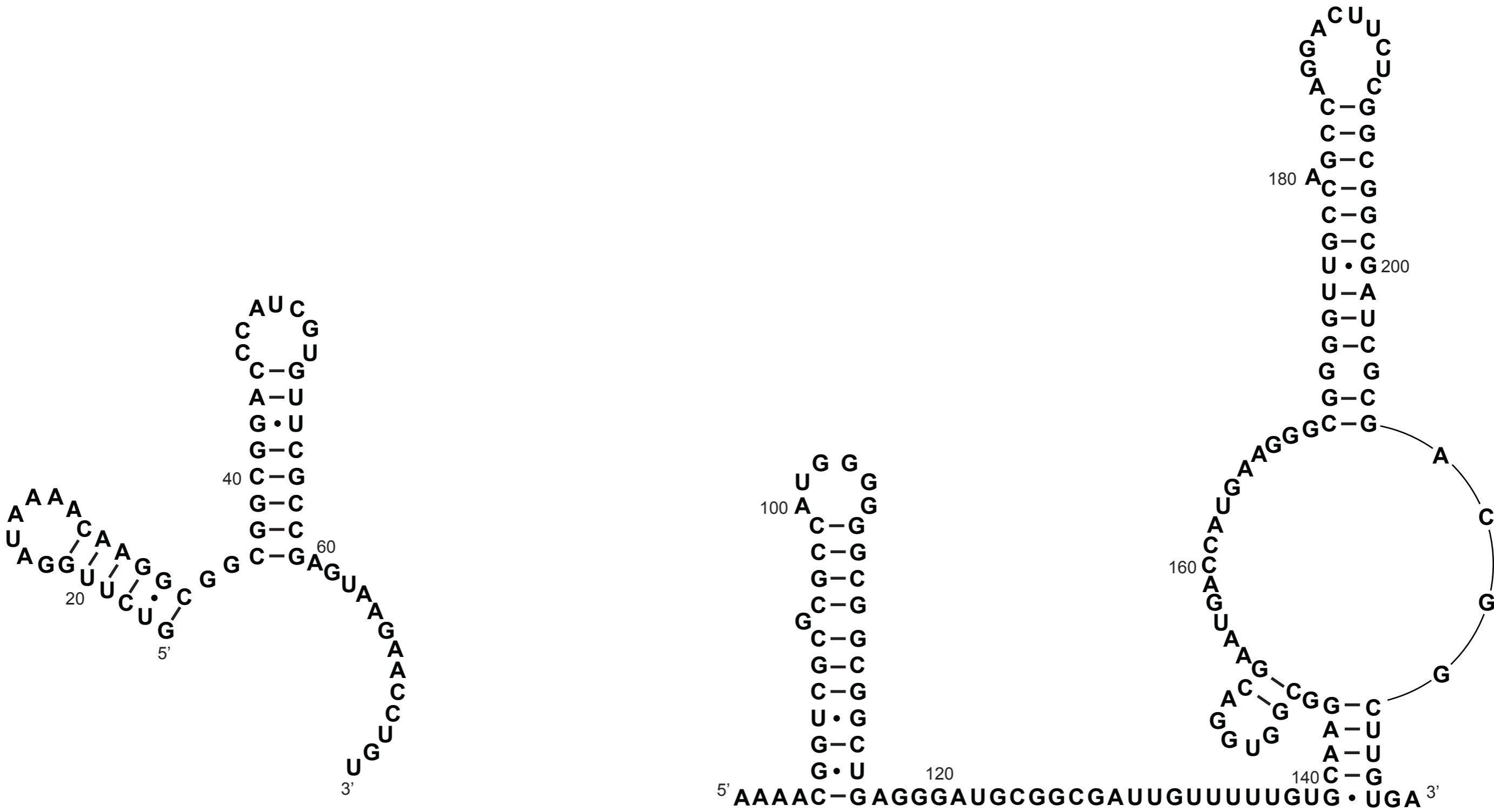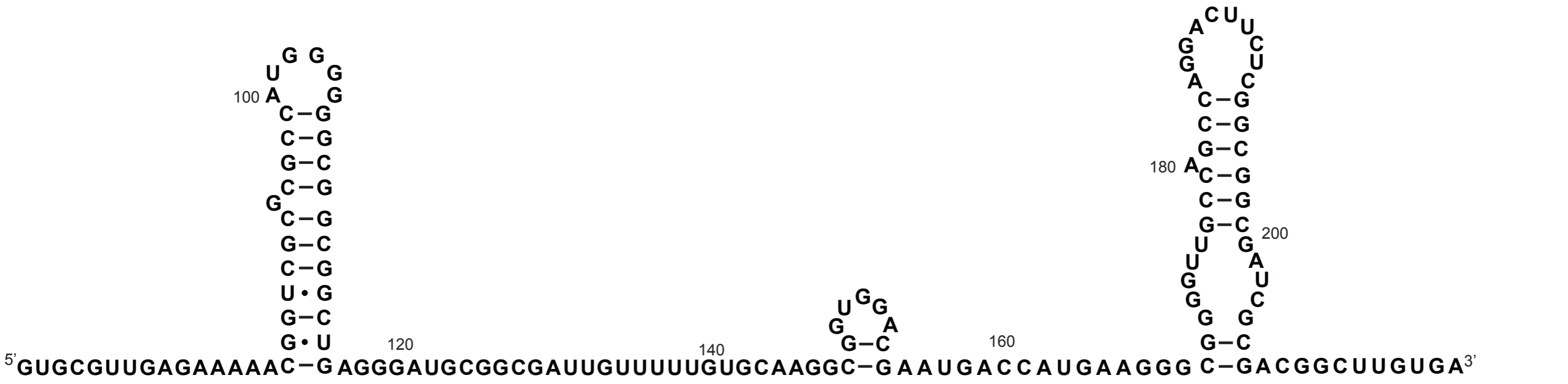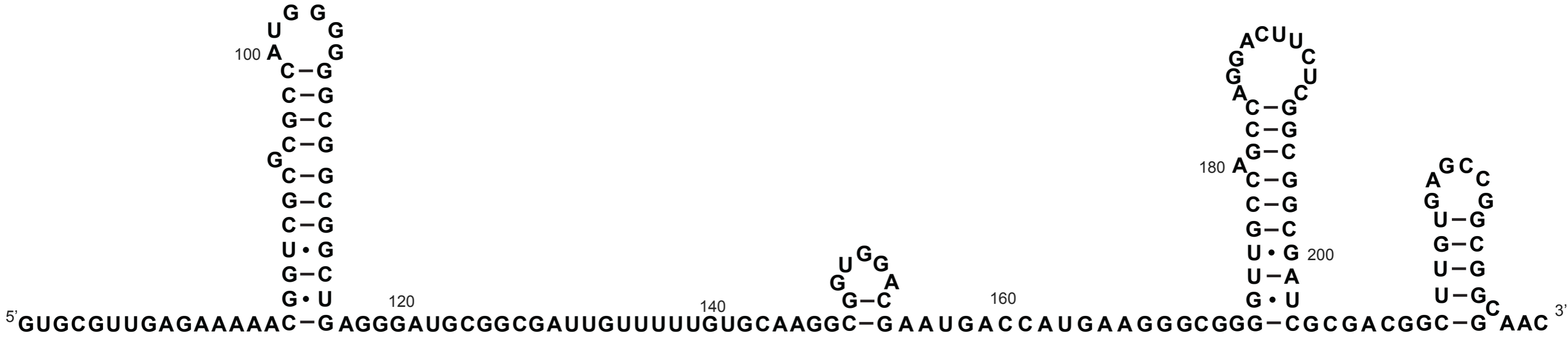

Supplement: Figure 1—figure supplement 2—source data 1. [file elife-71611-fig1-figsupp2-data1.zip › Figure 1-figure supplement 2-source data 1/panel B/CMfinder_results/subgroup1/combined.pdf]

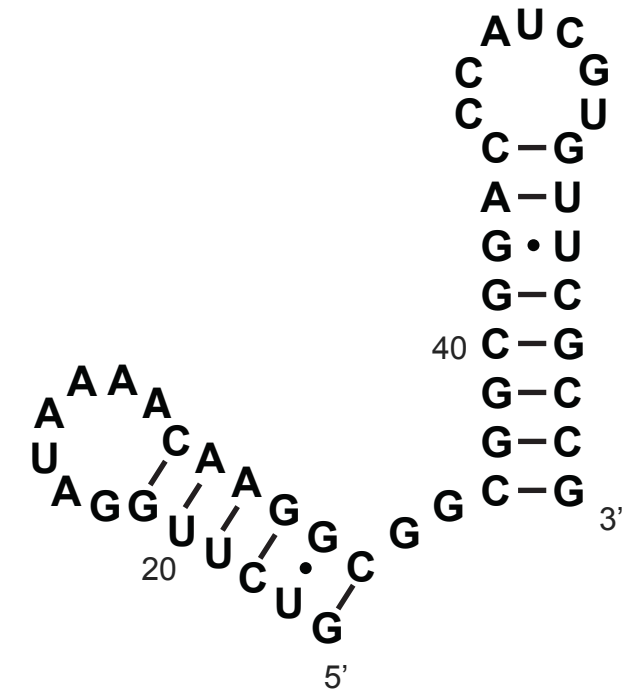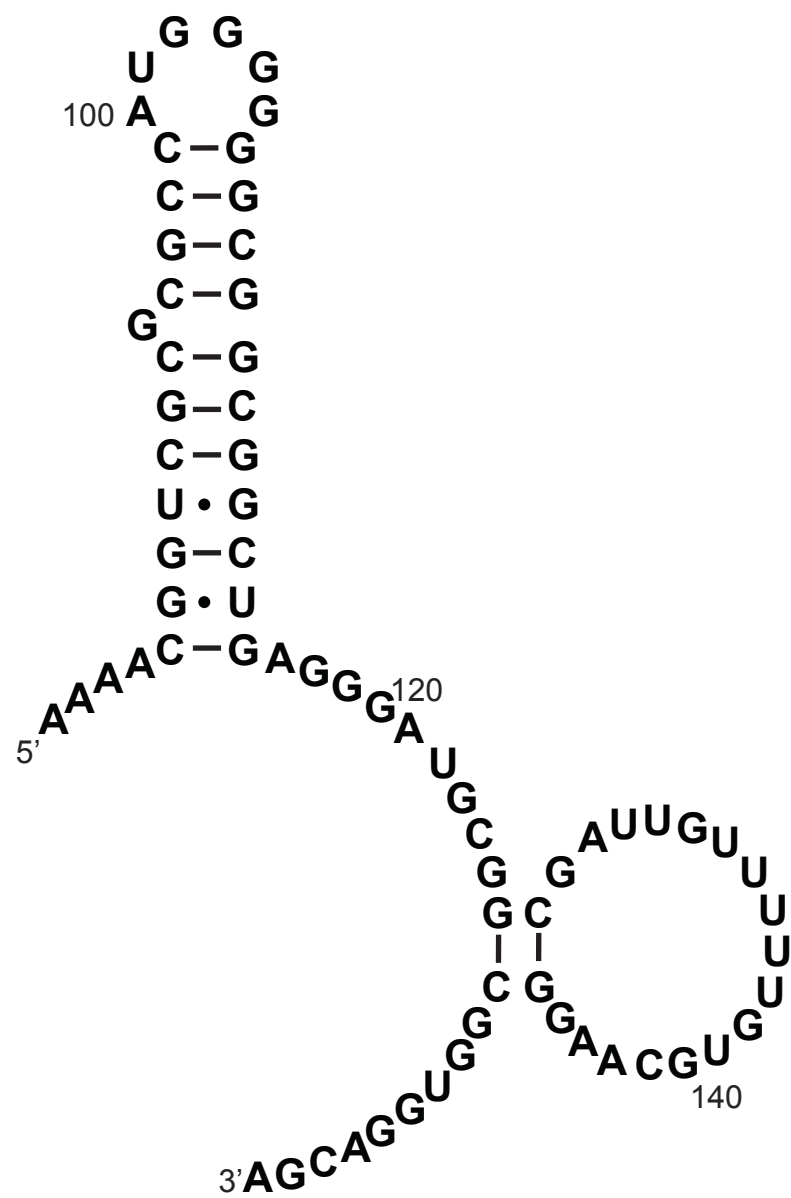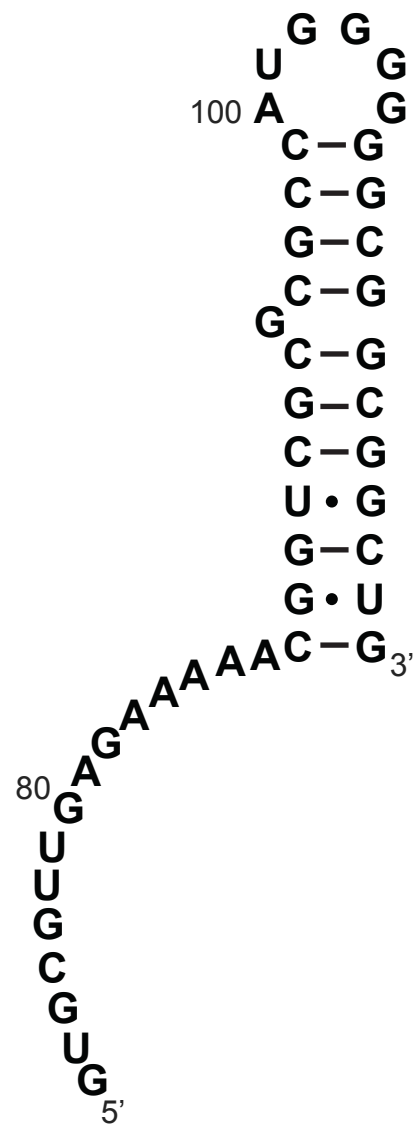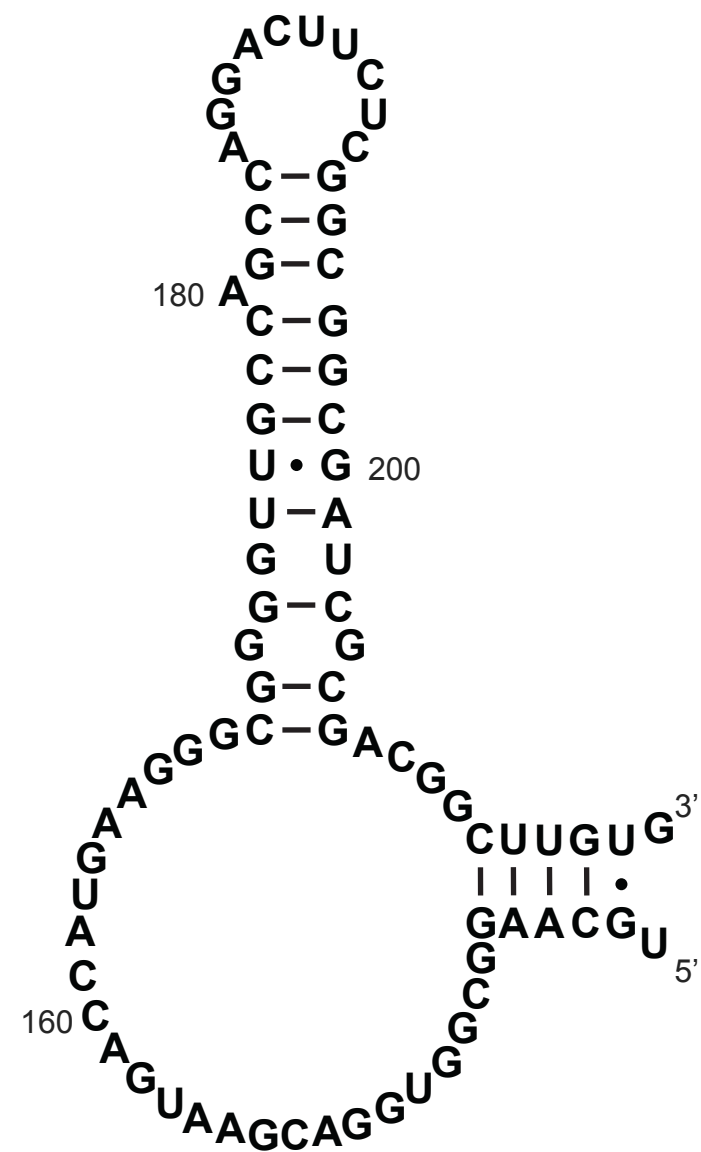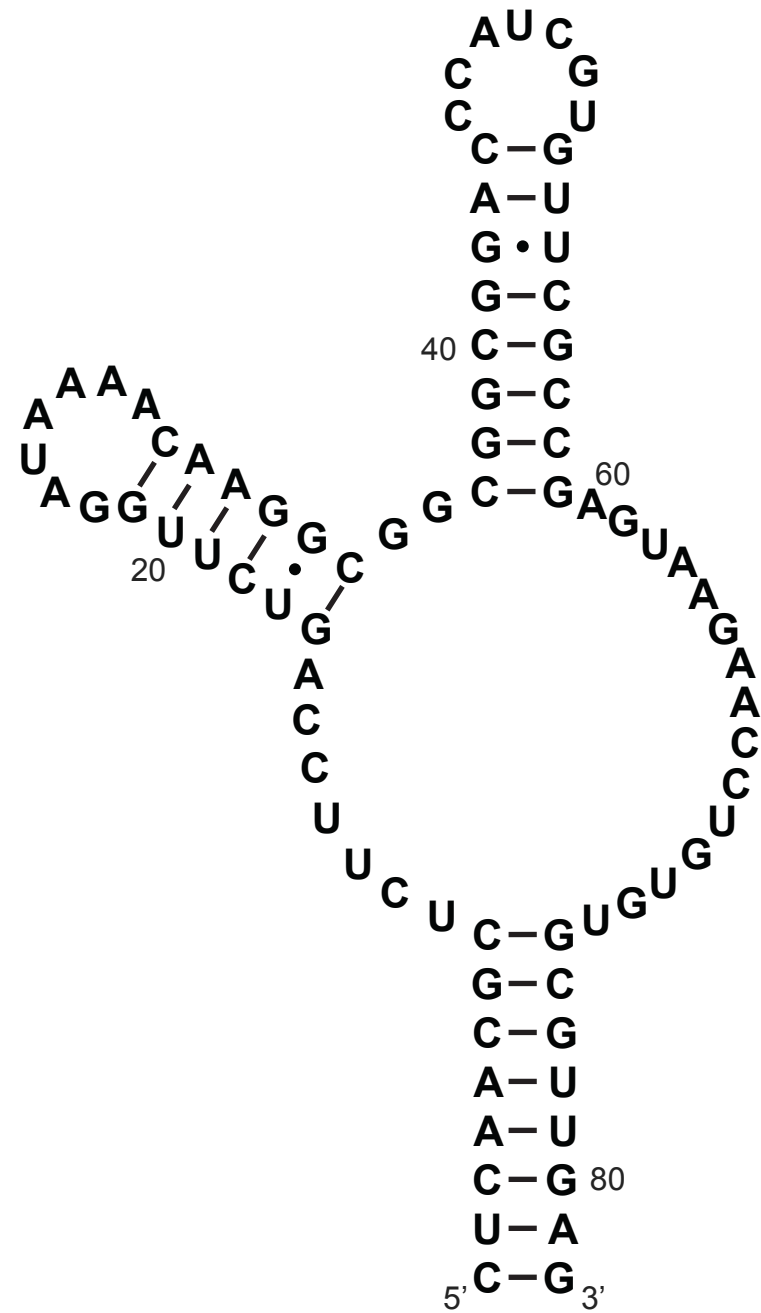

Supplement: Figure 1—figure supplement 2—source data 1. [file elife-71611-fig1-figsupp2-data1.zip › Figure 1-figure supplement 2-source data 1/panel B/CMfinder_results/subgroup1/h2.pdf]

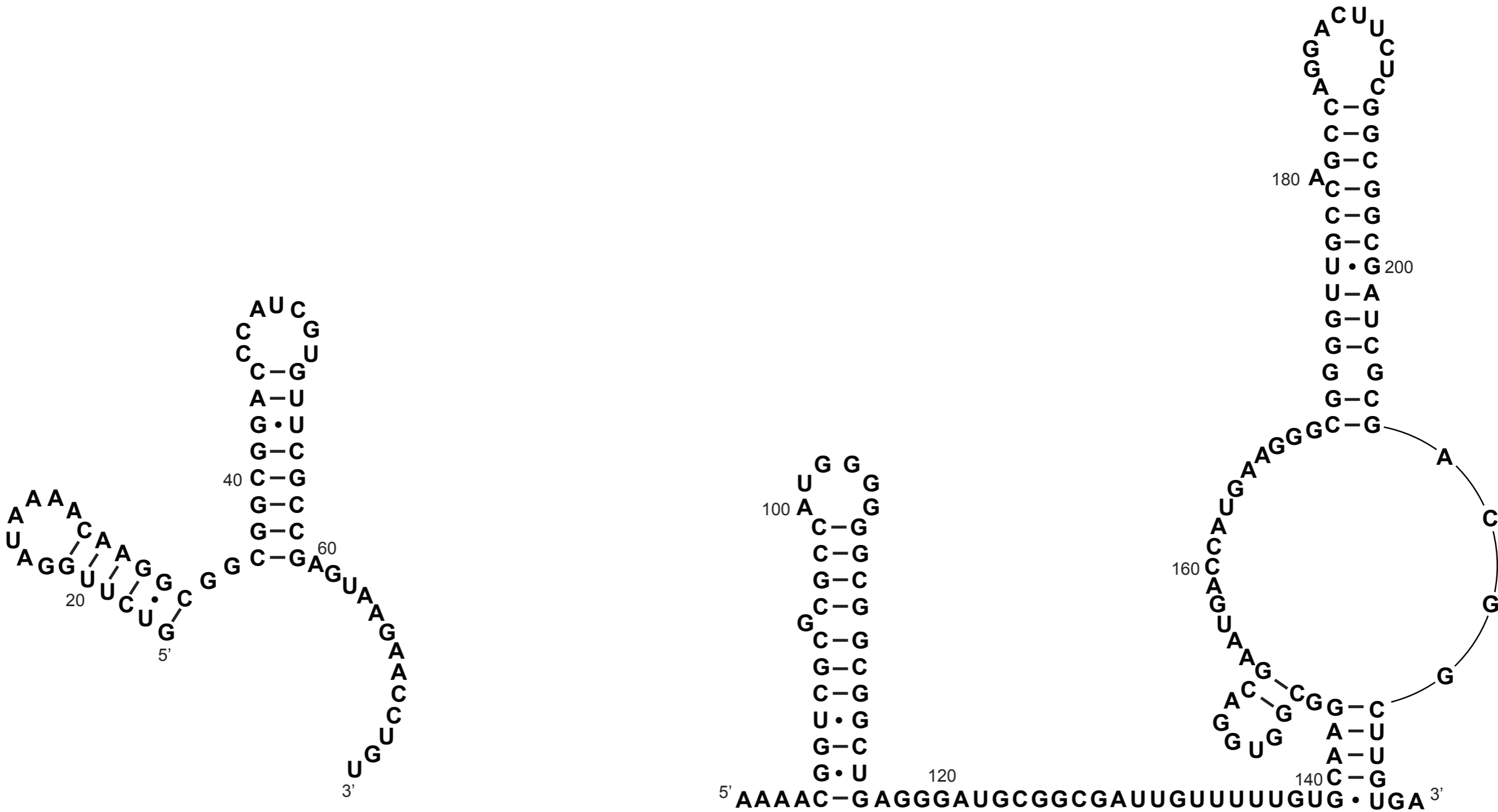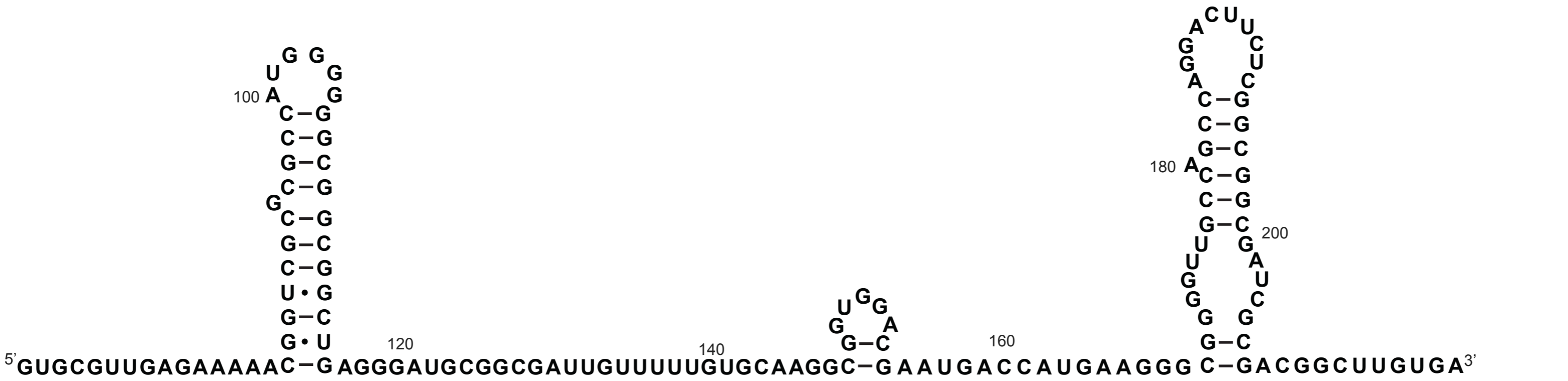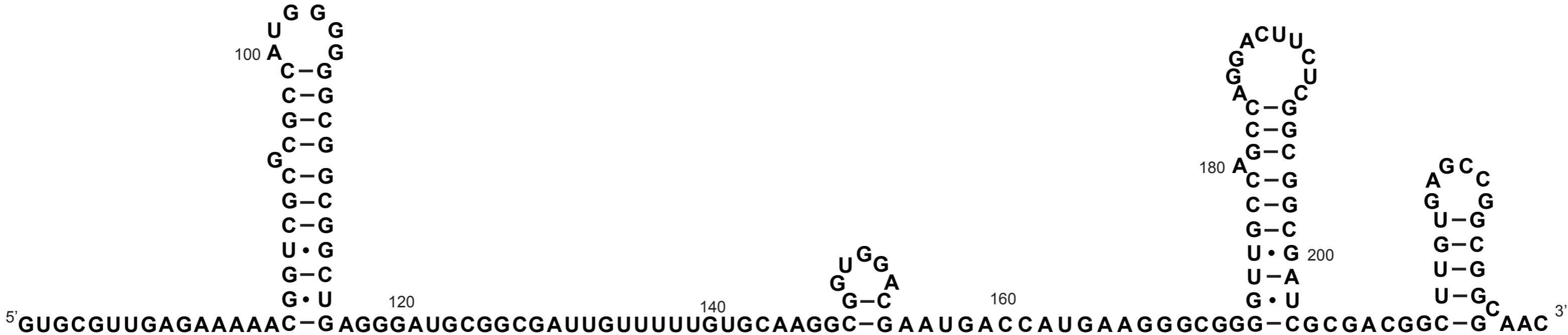

Supplement: Figure 1—figure supplement 2—source data 1. [file elife-71611-fig1-figsupp2-data1.zip › Figure 1-figure supplement 2-source data 1/panel B/CMfinder_results/subgroup2/combined.pdf]

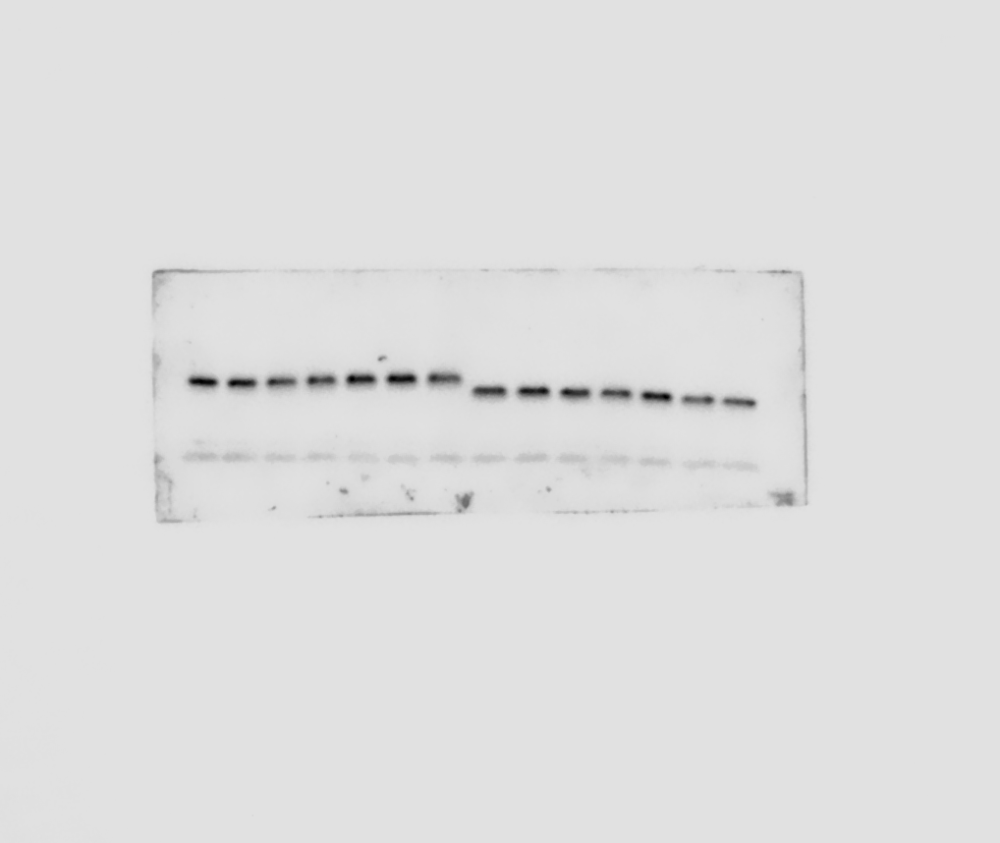

Supplement: Figure 4—figure supplement 2—source data 1. [file elife-71611-fig4-figsupp2-data1.zip › Figure 4-figure supplement 2-source data 1/panel A/Replicate2/WB_Nt_deltaNt_rep2.tif]

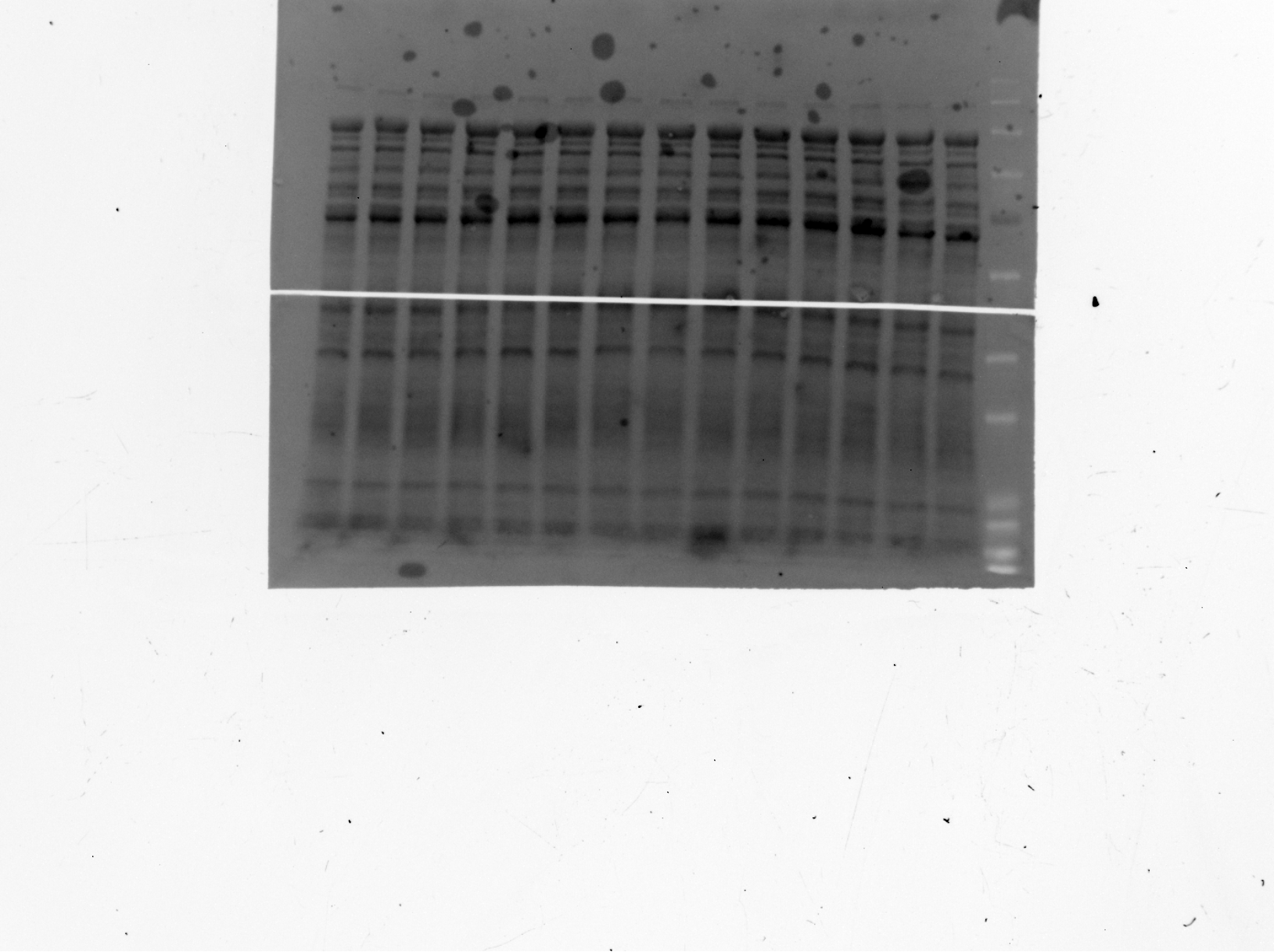

Supplement: Figure 4—figure supplement 2—source data 1. [file elife-71611-fig4-figsupp2-data1.zip › Figure 4-figure supplement 2-source data 1/panel A/Replicate2/Transfer_Nt_deltaNt_rep2.tif]

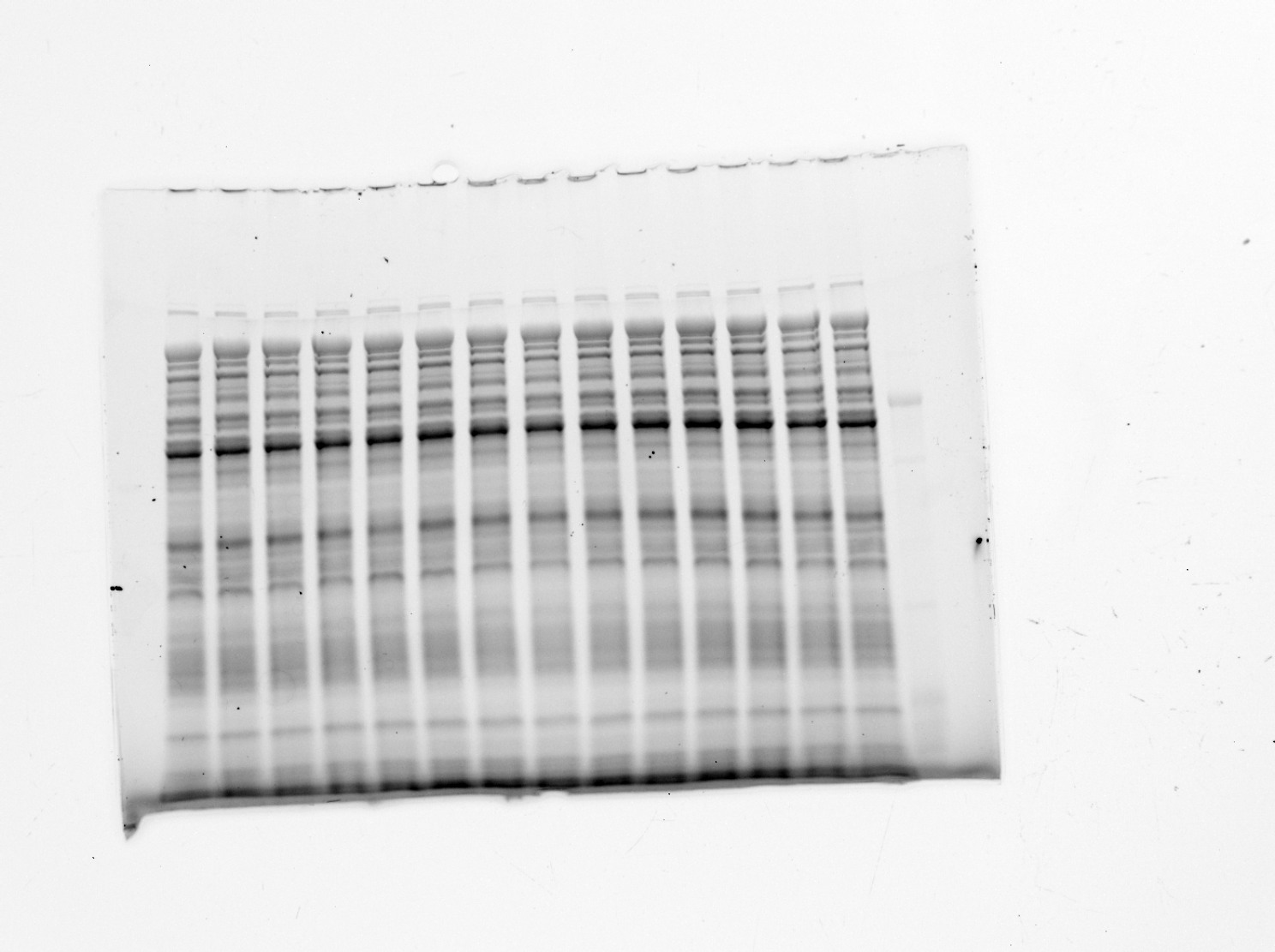

Supplement: Figure 4—figure supplement 2—source data 1. [file elife-71611-fig4-figsupp2-data1.zip › Figure 4-figure supplement 2-source data 1/panel A/Replicate2/PAGE_Nt_deltaNt_rep2.tif]

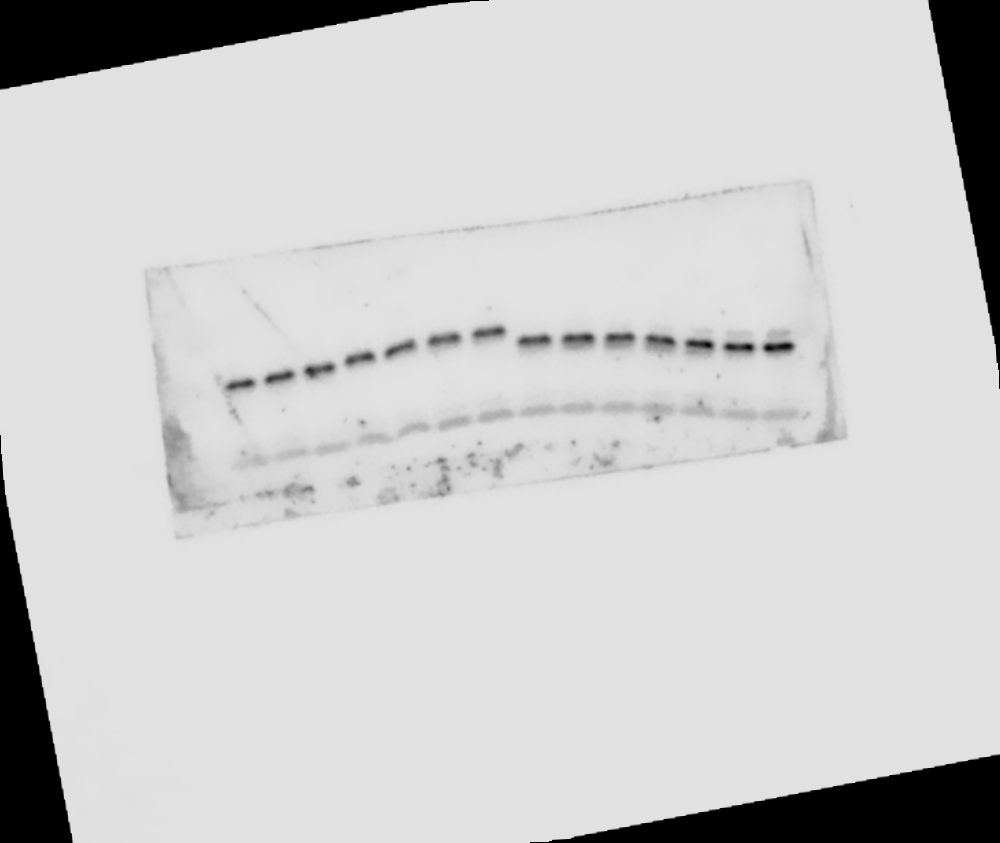

Supplement: Figure 4—figure supplement 2—source data 1. [file elife-71611-fig4-figsupp2-data1.zip › Figure 4-figure supplement 2-source data 1/panel A/Replicate3/WB_Nt_deltaNt_rep3.tif]

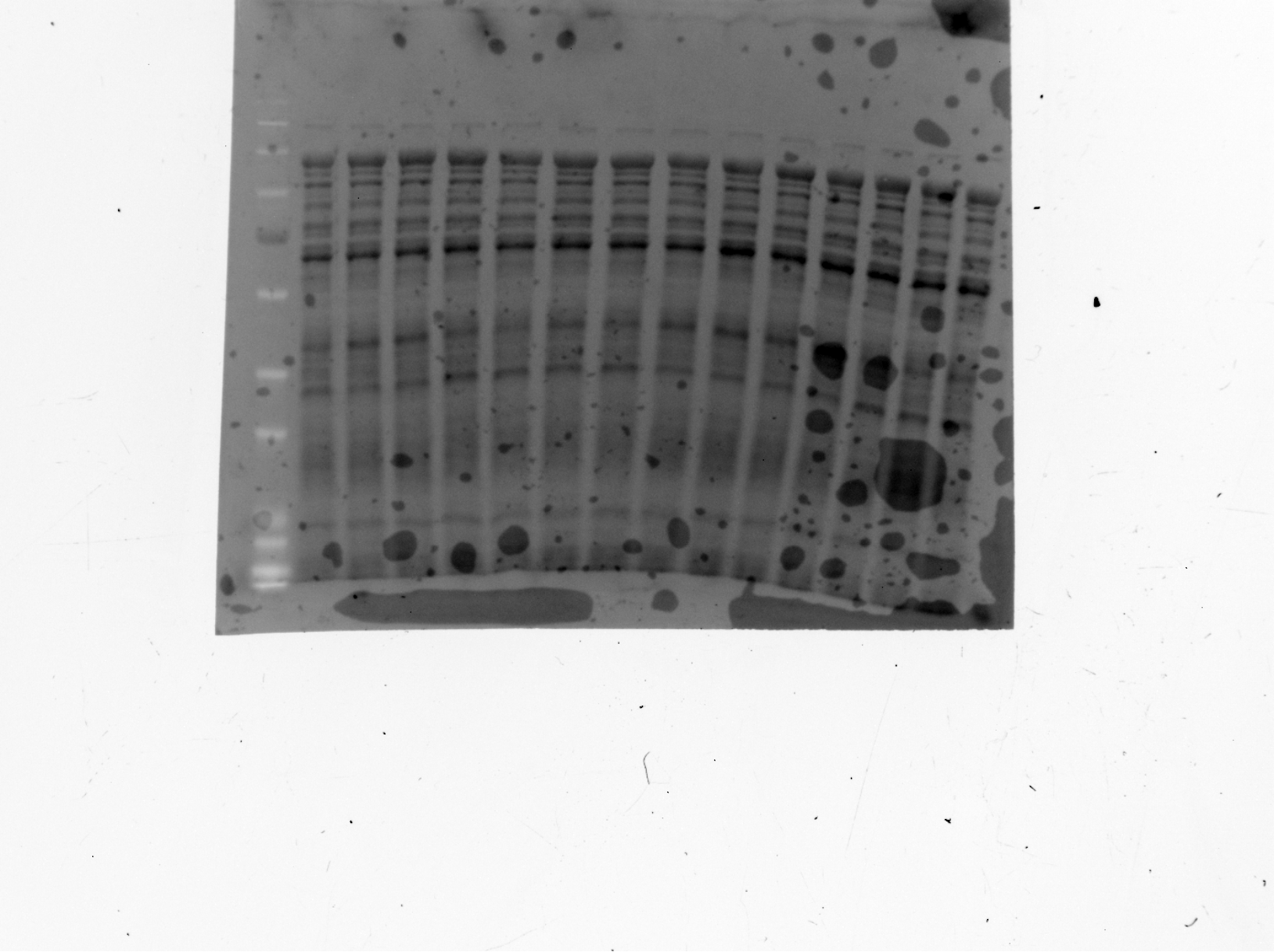

Supplement: Figure 4—figure supplement 2—source data 1. [file elife-71611-fig4-figsupp2-data1.zip › Figure 4-figure supplement 2-source data 1/panel A/Replicate3/Transfer_Nt_deltaNt_rep3.tif]

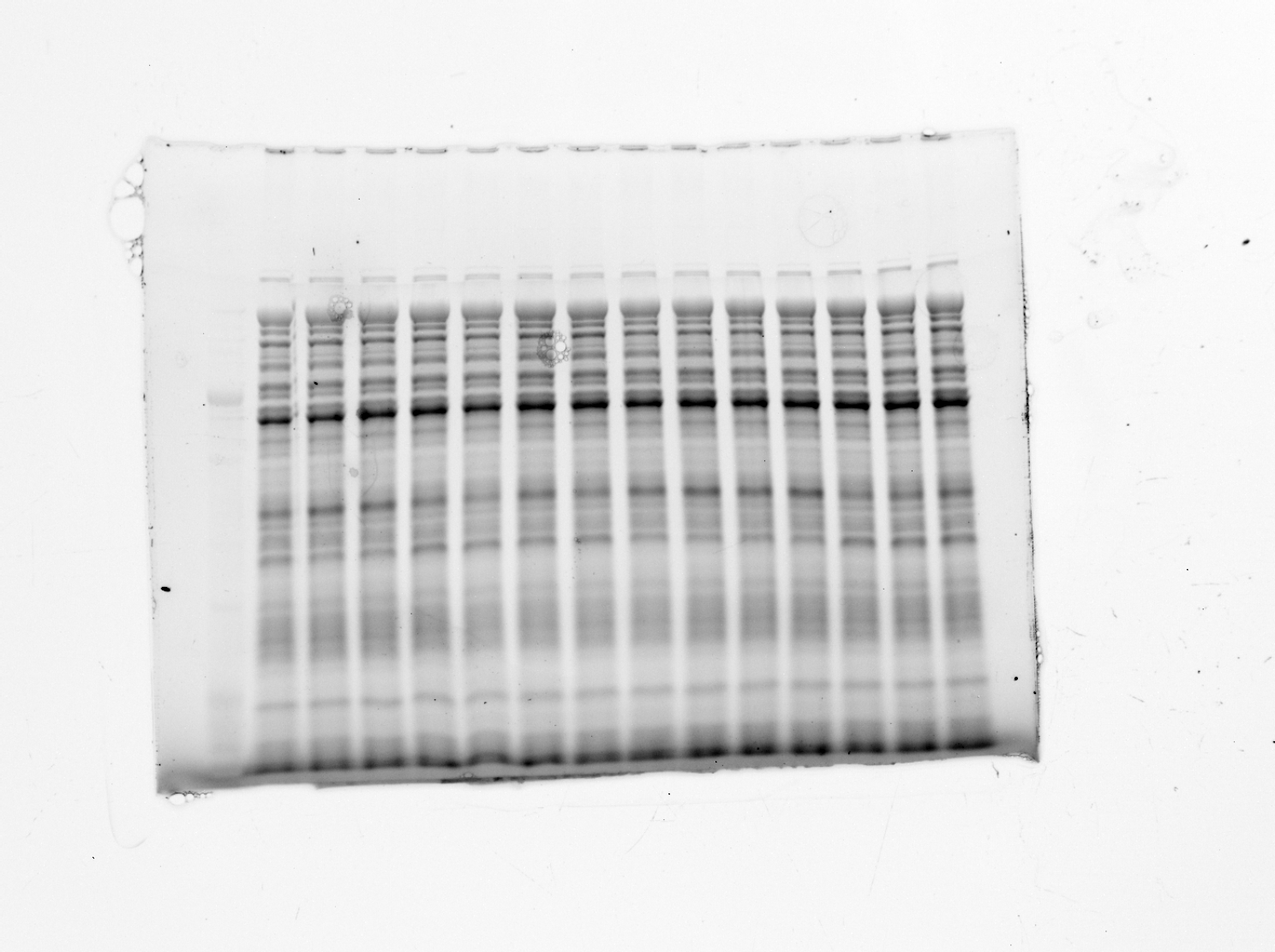

Supplement: Figure 4—figure supplement 2—source data 1. [file elife-71611-fig4-figsupp2-data1.zip › Figure 4-figure supplement 2-source data 1/panel A/Replicate3/PAGE_Nt_deltaNt_rep3.tif]

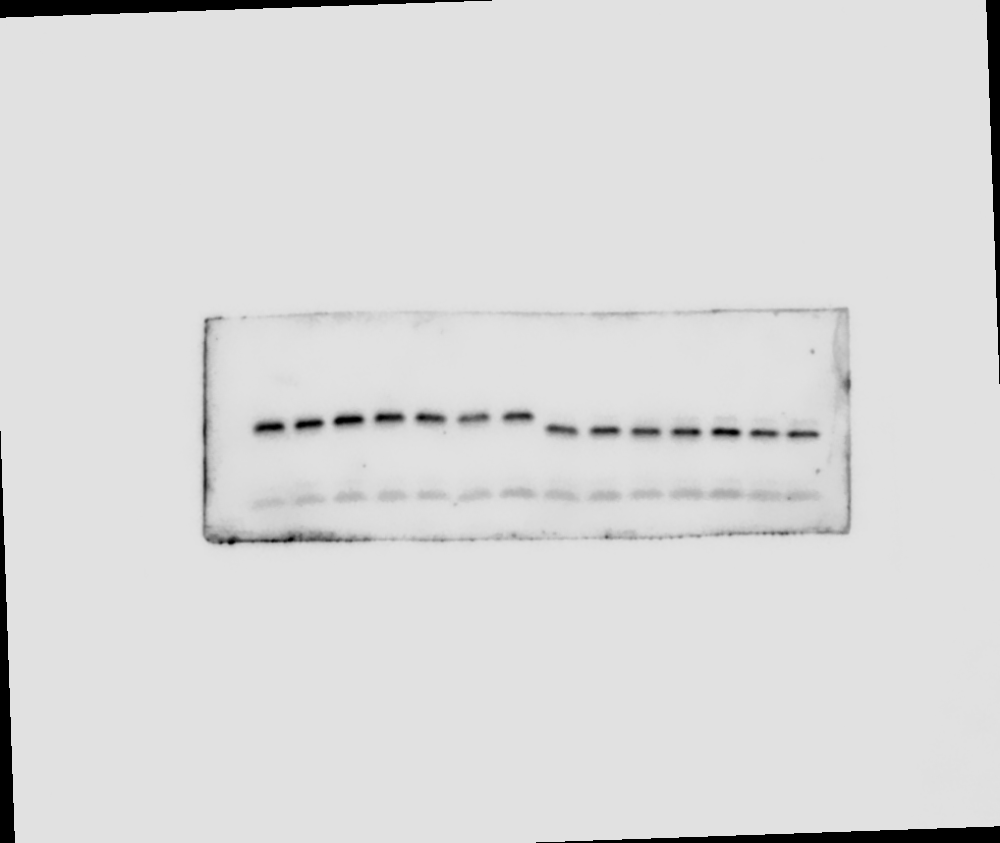

Supplement: Figure 4—figure supplement 2—source data 1. [file elife-71611-fig4-figsupp2-data1.zip › Figure 4-figure supplement 2-source data 1/panel A/Replicate1/WB_Nt_deltaNt_rep1.tif]

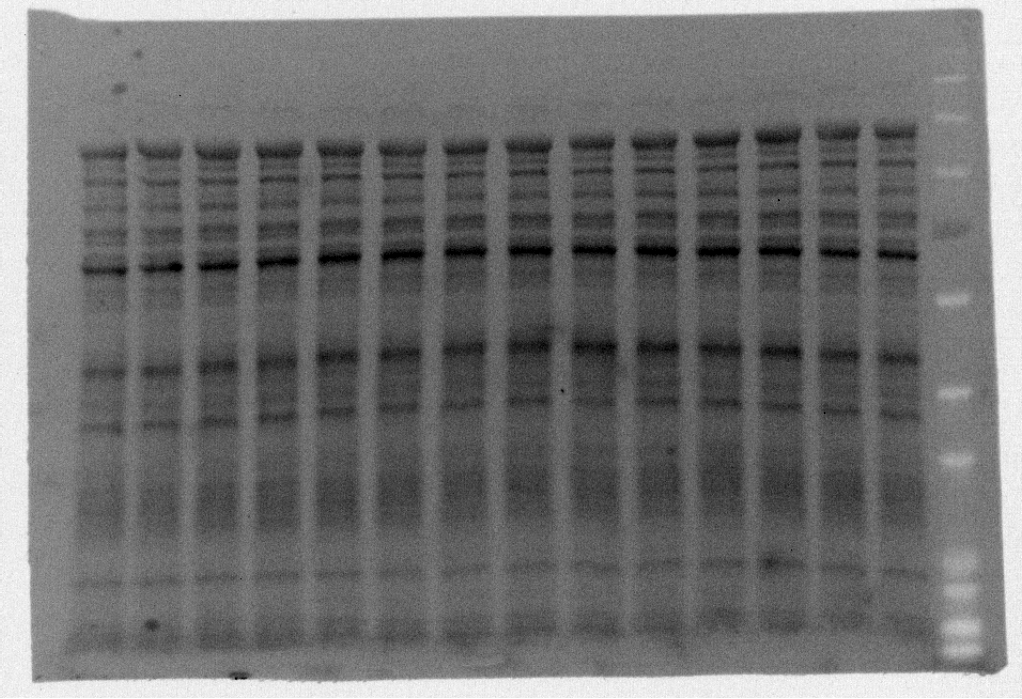

Supplement: Figure 4—figure supplement 2—source data 1. [file elife-71611-fig4-figsupp2-data1.zip › Figure 4-figure supplement 2-source data 1/panel A/Replicate1/Transfer_Nt_deltaNt_rep1.tif]

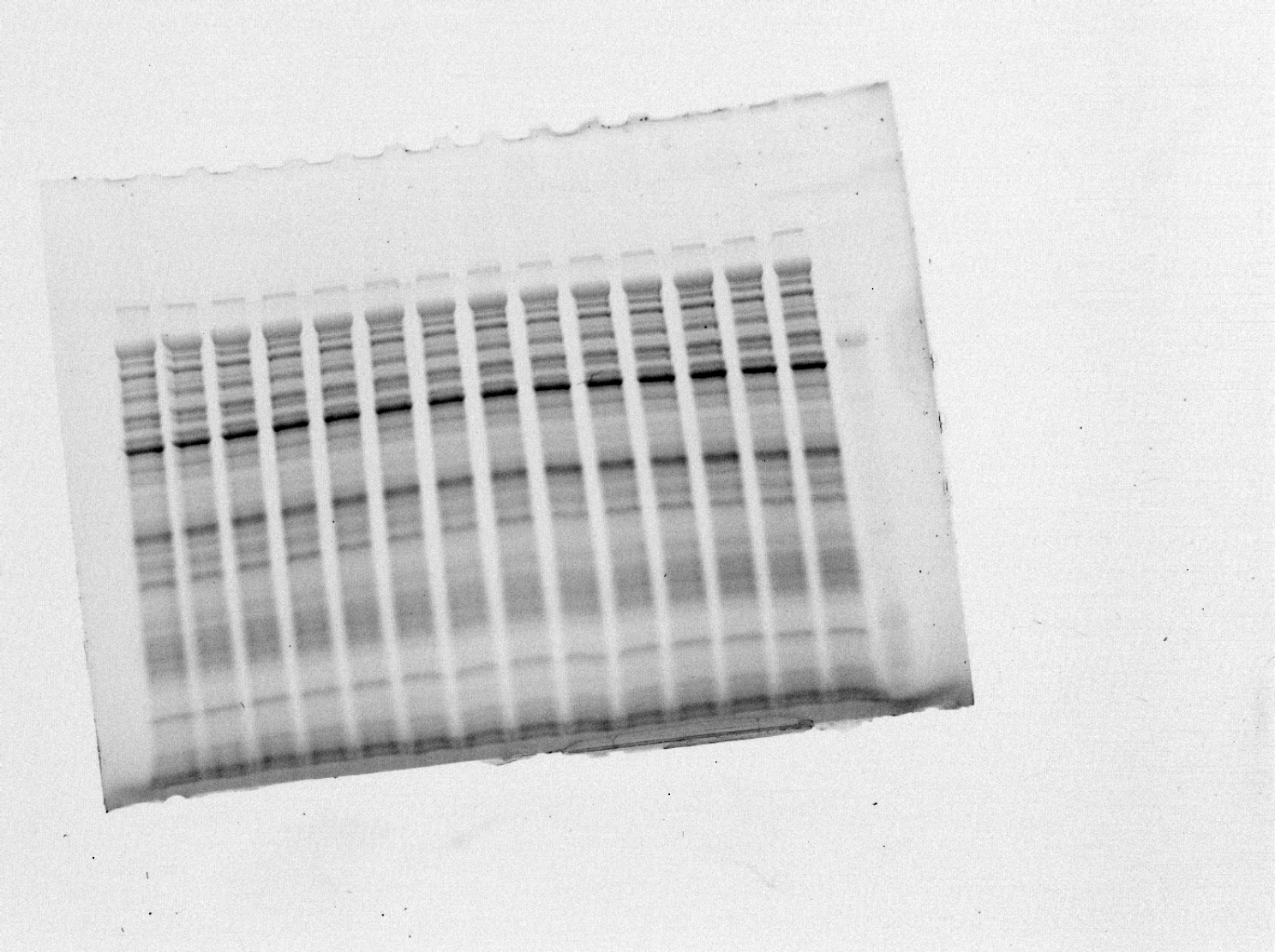

Supplement: Figure 4—figure supplement 2—source data 1. [file elife-71611-fig4-figsupp2-data1.zip › Figure 4-figure supplement 2-source data 1/panel A/Replicate1/PAGE_Nt_deltaNt_rep1.tif]
