# Supplementary material for: A nascent polypeptide sequence modulates DnaA translation elongation in response to nutrient availability
Source: eLife. 2021 Sep 15;10:e71611. doi: 10.7554/eLife.71611 (PMC8443254; doi:10.7554/eLife.71611)
Supplement: Supplementary file 1. [file elife-71611-supp1.docx]

**Supplementary file 1**

**pMR10 and pZE12 reporter plasmids construction**

**Supplementary file 1A. The pMR10-BG construct.** The pMR10-BG plasmid was derived from pMR10 (Roberts et al., 1996) by exchanging *lacZα* and *oriT* with a multicloning site. Grey: flanking sequences of the parental pMR10 plasmid. Red: HindIII and BamHI restriction sites.

**
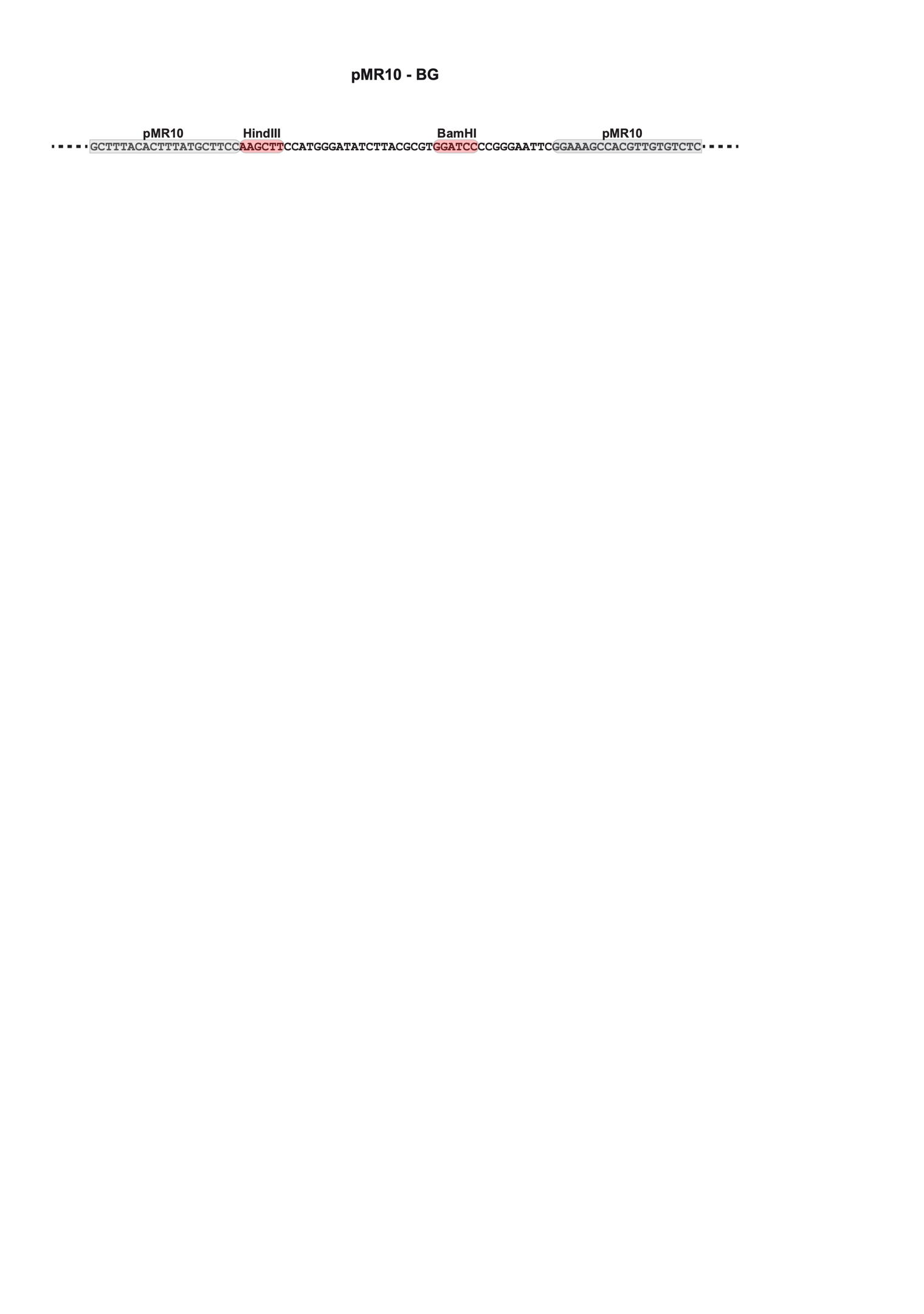
**

**Supplementary file 1B. The pMR10-*P_dnaA_*-5'UTR*_dnaA_*-N_t_-eGFP construct.** The pMR10-*P_dnaA_*-5'UTR*_dnaA_*-N_t_-eGFP reporter plasmid was derived upon digestion of pMR10-BG with HindIII and BamHI (restriction sites in red). The insert comprised, the *rrnB1*-T1T2 terminator (yellow), 245 bp upstream of the *dnaA* transcription start site (*P_dnaA_* - purple), the 5'UTR*_dnaA_* (blue), the first 78 bp of *dnaA* open reading frame (orange), the eGFP gene (green) and 63 bp downstream of the *dnaA* stop codon (3'UTR*_dnaA_* - pink).

**
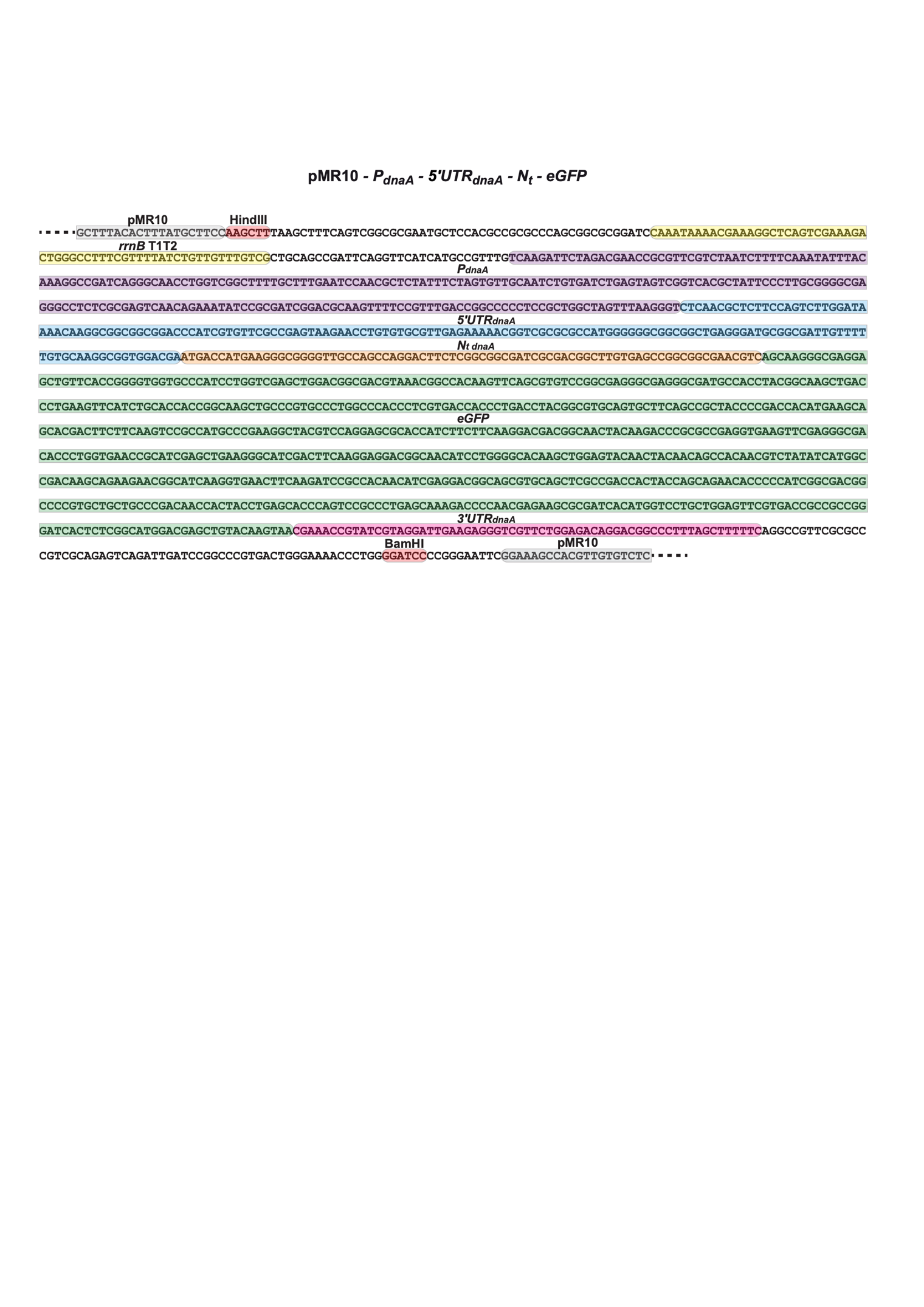
**

**Supplementary file 1C. The pZE12-BG construct.** Plasmid pZE12-BG was derived from the pZE12-luc plasmid by deleting the luciferase gene and part of the *P_λO1_* promoter. In grey, the flanking sequences of the original pZE12-luc plasmid.

**
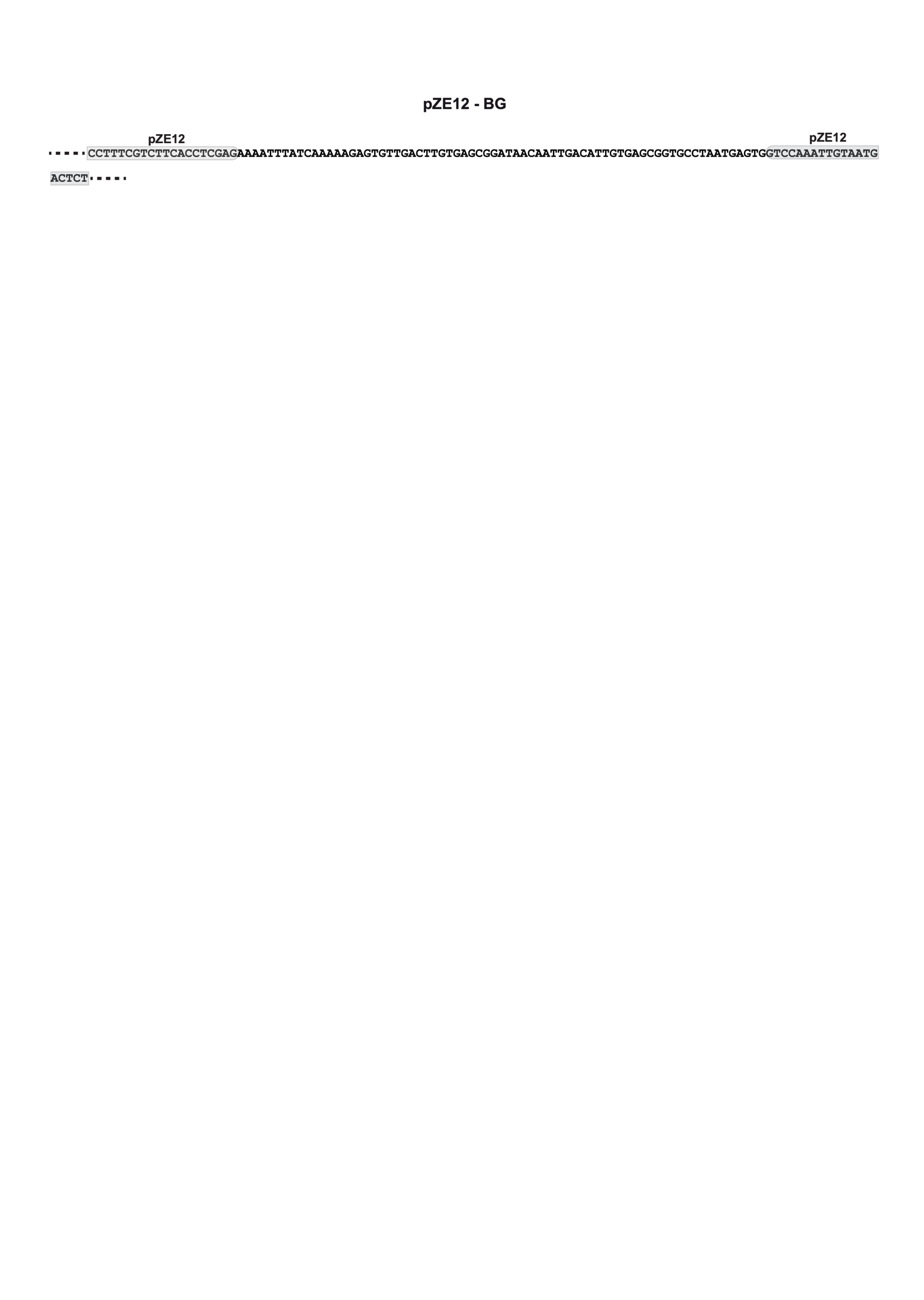
**

**Supplementary file 1D.** **The pZE12-*P*_λO1_-5'UTR*_dnaA_*-N_t_-eGFP construct.** The pZE12-*P*_λO1_-5'UTR*_dnaA_*-N_t_-eGFP reporter plasmid, was obtained from the pZE12-luc plasmid by exchanging the luciferase gene downstream of the Lambda O1 promoter (*P_λO1_* – purple) with an insert containing the 5'UTR*_dnaA_* (blue), the first 78 nt of *dnaA* open reading frame (orange), the eGFP gene (green) and the 3'UTR*_dnaA_* (pink).

**
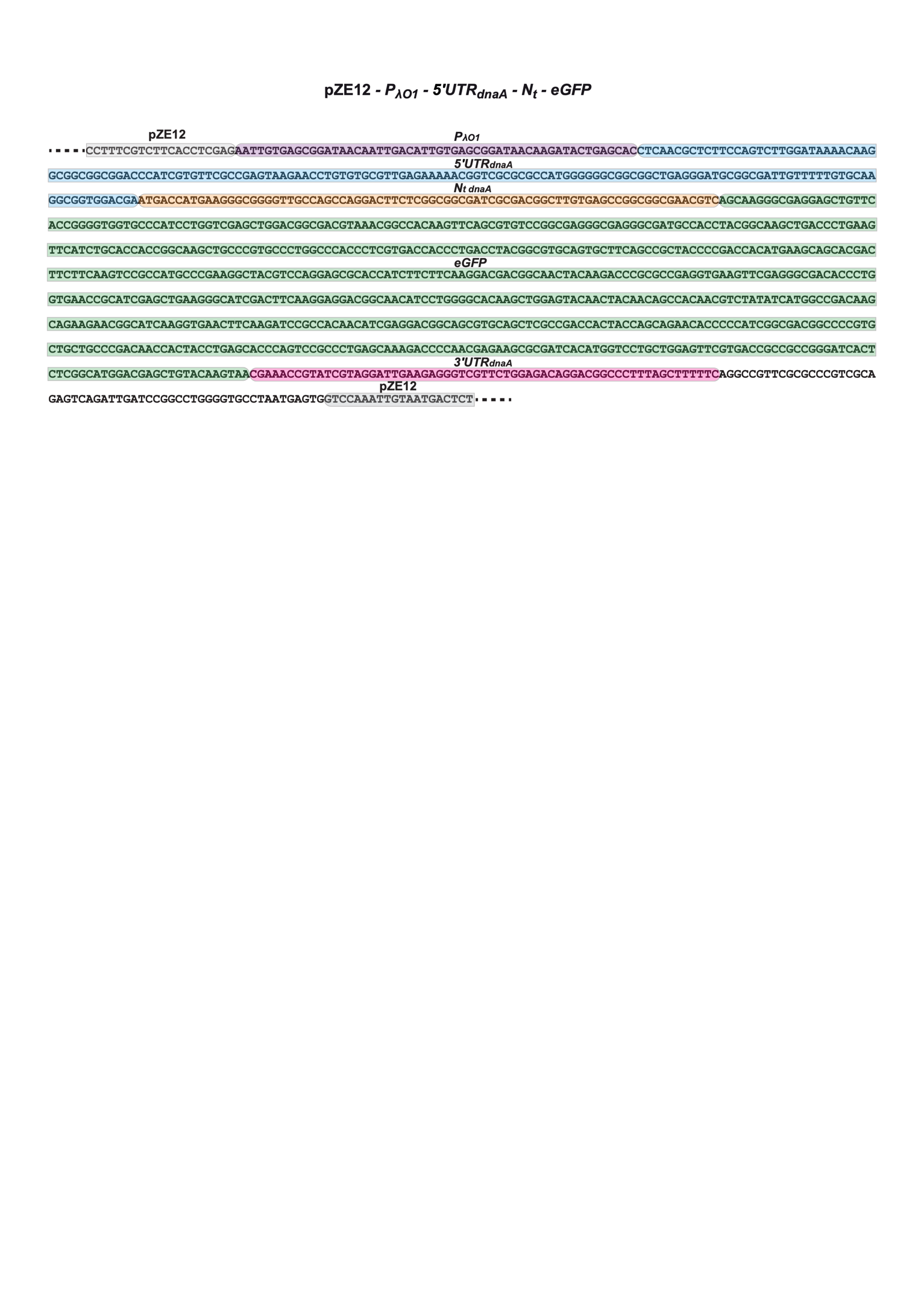
**

**Supplementary file 1E.** **Sequence of the 5**′**UTR and N_t_ modules in the reporter plasmid constructs.** The reporter plasmids were generated by site-directed mutagenesis, using the 5′UTR*_dnaA_*-N_t_ constructs as a PCR template (Supplementary file 1B and 1D). The sequences of the 5′UTR and N_t_ regions are shown in blue and orange respectively. Nucleotide substitutions and insertions are indicated in bold. Nucleotide deletions are indicated with the symbol “ – ”.

| **Strain** | **5′UTR-N_t_ sequence** |
| --- | --- |
| 5′UTR*_dnaA_*-N_t_ | CTCAACGCTCTTCCAGTCTTGGATAAAACAAGGCGGCGGCGGACCCATCGTGTTCGCCGAGTAAGAACCTGTGTGCGTTGAGAAAAACGGTCGCGCGCCATGGGGGGCGGCGGCTGAGGGATGCGGCGATTGTTTTTGTGCAAGGCGGTGGACGAATGACCATGAAGGGCGGGGTTGCCAGCCAGGACTTCTCGGCGGCGATCGCGACGGCTTGTGAGCCGGCGGCGAACGTC |
| 5′UTR*_dnaA_*-ΔN_t_ | CTCAACGCTCTTCCAGTCTTGGATAAAACAAGGCGGCGGCGGACCCATCGTGTTCGCCGAGTAAGAACCTGTGTGCGTTGAGAAAAACGGTCGCGCGCCATGGGGGGCGGCGGCTGAGGGATGCGGCGATTGTTTTTGTGCAAGGCGGTGGACGAATG------------------------------------------------------------------------GT**G** |
| 5′UTR*_6/13_*-N_t_ | **CACCAGGAGGAACAACAACT**ATGACCATGAAGGGCGGGGTTGCCAGCCAGGACTTCTCGGCGGCGATCGCGACGGCTTGTGAGCCGGCGGCGAACGTC |
| 5′UTR*_6/13_*-ΔN_t_ | **CACCAGGAGGAACAACAACT**ATG------------------------------------------------------------------------GT**G** |
| 5′UTR*_lac_*-N_t_ | **AATTGTGAGCGGATAACAATTTCACACAGGAAACAGCT**ATGACCATGAAGGGCGGGGTTGCCAGCCAGGACTTCTCGGCGGCGATCGCGACGGCTTGTGAGCCGGCGGCGAACGTC |
| 5′UTR*_lac_*-ΔN_t_ | **AATTGTGAGCGGATAACAATTTCACACAGGAAACAGCT**ATG------------------------------------------------------------------------GT**G** |
| G125C | CTCAACGCTCTTCCAGTCTTGGATAAAACAAGGCGGCGGCGGACCCATCGTGTTCGCCGAGTAAGAACCTGTGTGCGTTGAGAAAAACGGTCGCGCGCCATGGGGGGCGGCGGCTGAGGGATGC**C**GCGATTGTTTTTGTGCAAGGCGGTGGACGAATGACCATGAAGGGCGGGGTTGCCAGCCAGGACTTCTCGGCGGCGATCGCGACGGCTTGTGAGCCGGCGGCGAACGTC |
| C124A | CTCAACGCTCTTCCAGTCTTGGATAAAACAAGGCGGCGGCGGACCCATCGTGTTCGCCGAGTAAGAACCTGTGTGCGTTGAGAAAAACGGTCGCGCGCCATGGGGGGCGGCGGCTGAGGGATG**A**GGCGATTGTTTTTGTGCAAGGCGGTGGACGAATGACCATGAAGGGCGGGGTTGCCAGCCAGGACTTCTCGGCGGCGATCGCGACGGCTTGTGAGCCGGCGGCGAACGTC |
| G126A | CTCAACGCTCTTCCAGTCTTGGATAAAACAAGGCGGCGGCGGACCCATCGTGTTCGCCGAGTAAGAACCTGTGTGCGTTGAGAAAAACGGTCGCGCGCCATGGGGGGCGGCGGCTGAGGGATGCG**A**CGATTGTTTTTGTGCAAGGCGGTGGACGAATGACCATGAAGGGCGGGGTTGCCAGCCAGGACTTCTCGGCGGCGATCGCGACGGCTTGTGAGCCGGCGGCGAACGTC |
| C127A | CTCAACGCTCTTCCAGTCTTGGATAAAACAAGGCGGCGGCGGACCCATCGTGTTCGCCGAGTAAGAACCTGTGTGCGTTGAGAAAAACGGTCGCGCGCCATGGGGGGCGGCGGCTGAGGGATGCGG**A**GATTGTTTTTGTGCAAGGCGGTGGACGAATGACCATGAAGGGCGGGGTTGCCAGCCAGGACTTCTCGGCGGCGATCGCGACGGCTTGTGAGCCGGCGGCGAACGTC |
| U130A | CTCAACGCTCTTCCAGTCTTGGATAAAACAAGGCGGCGGCGGACCCATCGTGTTCGCCGAGTAAGAACCTGTGTGCGTTGAGAAAAACGGTCGCGCGCCATGGGGGGCGGCGGCTGAGGGATGCGGCGA**A**TGTTTTTGTGCAAGGCGGTGGACGAATGACCATGAAGGGCGGGGTTGCCAGCCAGGACTTCTCGGCGGCGATCGCGACGGCTTGTGAGCCGGCGGCGAACGTC |
| G132A | CTCAACGCTCTTCCAGTCTTGGATAAAACAAGGCGGCGGCGGACCCATCGTGTTCGCCGAGTAAGAACCTGTGTGCGTTGAGAAAAACGGTCGCGCGCCATGGGGGGCGGCGGCTGAGGGATGCGGCGATT**A**TTTTTGTGCAAGGCGGTGGACGAATGACCATGAAGGGCGGGGTTGCCAGCCAGGACTTCTCGGCGGCGATCGCGACGGCTTGTGAGCCGGCGGCGAACGTC |
| T1  (Δ2-81) | C--------------------------------------------------------------------------------GAAAAACGGTCGCGCGCCATGGGGGGCGGCGGCTGAGGGATGCGGCGATTGTTTTTGTGCA  AGGCGGTGGACGAATGACCATGAAGGGCGGGGTTGCCAGCCAGGACTTCTCGGCGGCGATCGCGACGGCTTGTGAGCCGGCGGCGAACGTC |
| T2  (Δ2-116) | C-------------------------------------------------------------------------------------------------------------------AGGGATGCGGCGATTGTTTTTGTGCA  AGGCGGTGGACGAATGACCATGAAGGGCGGGGTTGCCAGCCAGGACTTCTCGGCGGCGATCGCGACGGCTTGTGAGCCGGCGGCGAACGTC |
| T3  (Δ2-122) | C-------------------------------------------------------------------------------------------------------------------------GCGGCGATTGTTTTTGTGCA  AGGCGGTGGACGAATGACCATGAAGGGCGGGGTTGCCAGCCAGGACTTCTCGGCGGCGATCGCGACGGCTTGTGAGCCGGCGGCGAACGTC |
| T4  (Δ2-131) | C----------------------------------------------------------------------------------------------------------------------------------GTTTTTGTGCA  AGGCGGTGGACGAATGACCATGAAGGGCGGGGTTGCCAGCCAGGACTTCTCGGCGGCGATCGCGACGGCTTGTGAGCCGGCGGCGAACGTC |
| T5  (Δ2-137) | C----------------------------------------------------------------------------------------------------------------------------------------GTGCA  AGGCGGTGGACGAATGACCATGAAGGGCGGGGTTGCCAGCCAGGACTTCTCGGCGGCGATCGCGACGGCTTGTGAGCCGGCGGCGAACGTC |
| T6  (Δ2-140) | C-------------------------------------------------------------------------------------------------------------------------------------------CA  AGGCGGTGGACGAATGACCATGAAGGGCGGGGTTGCCAGCCAGGACTTCTCGGCGGCGATCGCGACGGCTTGTGAGCCGGCGGCGAACGTC |
| L1 | CTCAACGCTCTTCCAGTCTTGGATAAAACAAGGCGGCGGCGGACCCATCGTGTTCGCCGAGTAAGAACCTGTGTGCGTTGAGAAAAACGGTCGCGCGCCATGGGGGGCGGCGGCTGAGGGATGCGGCGATTG--**GAAA**--CA  AGGCGGTGGACGAATGACCATGAAGGGCGGGGTTGCCAGCCAGGACTTCTCGGCGGCGATCGCGACGGCTTGTGAGCCGGCGGCGAACGTC |
| L2 | CTCAACGCTCTTCCAGTCTTGGATAAAACAAGGCGGCGGCGGACCCATCGTGTTCGCCGAGTAAGAACCTGTGTGCGTTGAGAAAAACGGTCGCGCGCCATGGGGGGCGGCGGCTGAGGGATGCGGCGATTG--**CTCG**--CA  AGGCGGTGGACGAATGACCATGAAGGGCGGGGTTGCCAGCCAGGACTTCTCGGCGGCGATCGCGACGGCTTGTGAGCCGGCGGCGAACGTC |
| L3 | CTCAACGCTCTTCCAGTCTTGGATAAAACAAGGCGGCGGCGGACCCATCGTGTTCGCCGAGTAAGAACCTGTGTGCGTTGAGAAAAACGGTCGCGCGCCATGGGGGGCGGCGGCTGAGGGATGCGGCGATTG**AAAAA**GTGCAAGGCGGTGGACGAATGACCATGAAGGGCGGGGTTGCCAGCCAGGACTTCTCGGCGGCGATCGCGACGGCTTGTGAGCCGGCGGCGAACGTC |
| SCM1  (U157A-U163A) | CTCAACGCTCTTCCAGTCTTGGATAAAACAAGGCGGCGGCGGACCCATCGTGTTCGCCGAGTAAGAACCTGTGTGCGTTGAGAAAAACGGTCGCGCGCCATGGGGGGCGGCGGCTGAGGGATGCGGCGATTGTTTTTGTGCAAGGCGGTGGACGAA**A**GACCA**A**GAAGGGCGGGGTTGCCAGCCAGGACTTCTCGGCGGCGATCGCGACGGCTTGTGAGCCGGCGGCGAACGTC |
| SCM2  (G90U-C114A-U157A-U163A) | CTCAACGCTCTTCCAGTCTTGGATAAAACAAGGCGGCGGCGGACCCATCGTGTTCGCCGAGTAAGAACCTGTGTGCGTTGAGAAAAACG**T**TCGCGCGCCATGGGGGGCGGCGG**A**TGAGGGATGCGGCGATTGTTTTTGTGCAAGGCGGTGGACGAA**A**GACCA**A**GAAGGGCGGGGTTGCCAGCCAGGACTTCTCGGCGGCGATCGCGACGGCTTGTGAGCCGGCGGCGAACGTC |
| ΔP4  (Δ89-115) | CTCAACGCTCTTCCAGTCTTGGATAAAACAAGGCGGCGGCGGACCCATCGTGTTCGCCGAGTAAGAACCTGTGTGCGTTGAGAAAAAC---------------------------GAGGGATGCGGCGATTGTTTTTGTGCA  AGGCGGTGGACGAATGACCATGAAGGGCGGGGTTGCCAGCCAGGACTTCTCGGCGGCGATCGCGACGGCTTGTGAGCCGGCGGCGAACGTC |
| ΔP5  (Δ123-152) | CTCAACGCTCTTCCAGTCTTGGATAAAACAAGGCGGCGGCGGACCCATCGTGTTCGCCGAGTAAGAACCTGTGTGCGTTGAGAAAAACGGTCGCGCGCCATGGGGGGCGGCGGCTGAGGGAT------------------------------CGAATGACCATGAAGGGCGGGGTTGCCAGCCAGGACTTCTCGGCGGCGATCGCGACGGCTT  GTGAGCCGGCGGCGAACGTC |
| 2 x N_t_ | CTCAACGCTCTTCCAGTCTTGGATAAAACAAGGCGGCGGCGGACCCATCGTGTTCGCCGAGTAAGAACCTGTGTGCGTTGAGAAAAACGGTCGCGCGCCATGGGGGGCGGCGGCTGAGGGATGCGGCGATTGTTTTTGTGCAAGGCGGTGGACGAATGACCATGAAGGGCGGGGTTGCCAGCCAGGACTTCTCGGCGGCGATCGCGACGGCTTGTGAGCCGGCGGCGAACGTC**ACCATGAAGGGCGGGGTTGCCAGCCAGGACTTCTCGGCGGCGATCGCGACGGCTTGTGAGCCGGCGGCGAACGTC** |
| dfsN_t_ | CTCAACGCTCTTCCAGTCTTGGATAAAACAAGGCGGCGGCGGACCCATCGTGTTCGCCGAGTAAGAACCTGTGTGCGTTGAGAAAAACGGTCGCGCGCCATGGGGGGCGGCGGCTGAGGGATGCGGCGATTGTTTTTGTGCAAGGCGGTGGACGAATGACCAT-AAGGGCGGGGTTGCCAGCCAGGACTTCTCGGCGGCGATCGCGACGGCTT  GTGAGCCGGCGGCGAACGTC**G** |
| dfsN_t_ + N_t_ | CTCAACGCTCTTCCAGTCTTGGATAAAACAAGGCGGCGGCGGACCCATCGTGTTCGCCGAGTAAGAACCTGTGTGCGTTGAGAAAAACGGTCGCGCGCCATGGGGGGCGGCGGCTGAGGGATGCGGCGATTGTTTTTGTGCAAGGCGGTGGACGAATGACCAT-AAGGGCGGGGTTGCCAGCCAGGACTTCTCGGCGGCGATCGCGACGGCTT  GTGAGCCGGCGGCGAACGTC**GACCATGAAGGGCGGGGTTGCCAGCCAGGACTTCTCGGCGGCGATCGCGACGGCTTGTGAGCCGGCGGCGAACGTC** |
| mut D1  (Δ171-200) | CTCAACGCTCTTCCAGTCTTGGATAAAACAAGGCGGCGGCGGACCCATCGTGTTCGCCGAGTAAGAACCTGTGTGCGTTGAGAAAAACGGTCGCGCGCCATGGGGGGCGGCGGCTGAGGGATGCGGCGATTGTTTTTGTGCAAGGCGGTGGACGAATGACCATGAAGGGC------------------------------ATCGCGACGGCTT  GTGAGCCGGCGGCGAACGTC |
| mut D2  (Δ207-221) | CTCAACGCTCTTCCAGTCTTGGATAAAACAAGGCGGCGGCGGACCCATCGTGTTCGCCGAGTAAGAACCTGTGTGCGTTGAGAAAAACGGTCGCGCGCCATGGGGGGCGGCGGCTGAGGGATGCGGCGATTGTTTTTGTGCAAGGCGGTGGACGAATGACCATGAAGGGCGGGGTTGCCAGCCAGGACTTCTCGGCGGCGATCGCG-------  --------GCGGCGAACGTC |
| ΔAAI | CTCAACGCTCTTCCAGTCTTGGATAAAACAAGGCGGCGGCGGACCCATCGTGTTCGCCGAGTAAGAACCTGTGTGCGTTGAGAAAAACGGTCGCGCGCCATGGGGGGCGGCGGCTGAGGGATGCGGCGATTGTTTTTGTGCAAGGCGGTGGACGAATGACCATGAAGGGCGGGGTTGCCAGCCAGGACTTCTCG---------GCGACGGCTT  GTGAGCCGGCGGCGAACGTC |
| AAI → DDK | CTCAACGCTCTTCCAGTCTTGGATAAAACAAGGCGGCGGCGGACCCATCGTGTTCGCCGAGTAAGAACCTGTGTGCGTTGAGAAAAACGGTCGCGCGCCATGGGGGGCGGCGGCTGAGGGATGCGGCGATTGTTTTTGTGCAAGGCGGTGGACGAATGACCATGAAGGGCGGGGTTGCCAGCCAGGACTTCTCG**GACGACAAG**GCGACGGCTTGTGAGCCGGCGGCGAACGTC |
| mut CM1 | CTCAACGCTCTTCCAGTCTTGGATAAAACAAGGCGGCGGCGGACCCATCGTGTTCGCCGAGTAAGAACCTGTGTGCGTTGAGAAAAACGGTCGCGCGCCATGGGGGGCGGCGGCTGAGGGATGCGGCGATTGTTTTTGTGCAAGGCGGTGGACGAATGACCATGAAGGGCGGGGTTGCCAGCCAGGACTTCTCGGC**A**GC**A**AT**A**GCGACGGCTTGTGAGCCGGCGGCGAACGTC |
| mut CM2 | CTCAACGCTCTTCCAGTCTTGGATAAAACAAGGCGGCGGCGGACCCATCGTGTTCGCCGAGTAAGAACCTGTGTGCGTTGAGAAAAACGGTCGCGCGCCATGGGGGGCGGCGGCTGAGGGATGCGGCGATTGTTTTTGTGCAAGGCGGTGGACGAATGACCATGAAGGGCGGGGTTGCCAGCCAGGACTTCTCGGC**C**GC**C**ATCGCGACGGCTTGTGAGCCGGCGGCGAACGTC |
| mut 1  (KGG → ADD) | CTCAACGCTCTTCCAGTCTTGGATAAAACAAGGCGGCGGCGGACCCATCGTGTTCGCCGAGTAAGAACCTGTGTGCGTTGAGAAAAACGGTCGCGCGCCATGGGGGGCGGCGGCTGAGGGATGCGGCGATTGTTTTTGTGCAAGGCGGTGGACGAATGACCATG**GCGGACGAC**GTTGCCAGCCAGGACTTCTCGGCGGCGATCGCGACGGCTTGTGAGCCGGCGGCGAACGTC |
| mut 2  (ΔKGG) | CTCAACGCTCTTCCAGTCTTGGATAAAACAAGGCGGCGGCGGACCCATCGTGTTCGCCGAGTAAGAACCTGTGTGCGTTGAGAAAAACGGTCGCGCGCCATGGGGGGCGGCGGCTGAGGGATGCGGCGATTGTTTTTGTGCAAGGCGGTGGACGAATGACCATG---------GTTGCCAGCCAGGACTTCTCGGCGGCGATCGCGACGGCTT  GTGAGCCGGCGGCGAACGTC |
| mut 3  (ΔKGGVA) | CTCAACGCTCTTCCAGTCTTGGATAAAACAAGGCGGCGGCGGACCCATCGTGTTCGCCGAGTAAGAACCTGTGTGCGTTGAGAAAAACGGTCGCGCGCCATGGGGGGCGGCGGCTGAGGGATGCGGCGATTGTTTTTGTGCAAGGCGGTGGACGAATGACCATG---------------AGCCAGGACTTCTCGGCGGCGATCGCGACGGCTT  GTGAGCCGGCGGCGAACGTC |
| mut 4  (ΔGV) | CTCAACGCTCTTCCAGTCTTGGATAAAACAAGGCGGCGGCGGACCCATCGTGTTCGCCGAGTAAGAACCTGTGTGCGTTGAGAAAAACGGTCGCGCGCCATGGGGGGCGGCGGCTGAGGGATGCGGCGATTGTTTTTGTGCAAGGCGGTGGACGAATGACCATGAAGGGC------GCCAGCCAGGACTTCTCGGCGGCGATCGCGACGGCTT  GTGAGCCGGCGGCGAACGTC |
| mut 5  (ΔQ10 + ΔS13) | CTCAACGCTCTTCCAGTCTTGGATAAAACAAGGCGGCGGCGGACCCATCGTGTTCGCCGAGTAAGAACCTGTGTGCGTTGAGAAAAACGGTCGCGCGCCATGGGGGGCGGCGGCTGAGGGATGCGGCGATTGTTTTTGTGCAAGGCGGTGGACGAATGACCATGAAGGGCGGGGTTGCCAGC---GACTTC---GCGGCGATCGCGACGGCTT  GTGAGCCGGCGGCGAACGTC |
| mut 6  (DF → LN) | CTCAACGCTCTTCCAGTCTTGGATAAAACAAGGCGGCGGCGGACCCATCGTGTTCGCCGAGTAAGAACCTGTGTGCGTTGAGAAAAACGGTCGCGCGCCATGGGGGGCGGCGGCTGAGGGATGCGGCGATTGTTTTTGTGCAAGGCGGTGGACGAATGACCATGAAGGGCGGGGTTGCCAGCCAG**CTCAAC**TCGGCGGCGATCGCGACGGCTTGTGAGCCGGCGGC |
