# Supplementary material for: A nascent polypeptide sequence modulates DnaA translation elongation in response to nutrient availability
Source: eLife. 2021 Sep 15;10:e71611. doi: 10.7554/eLife.71611 (PMC8443254; doi:10.7554/eLife.71611)
Supplement: Supplementary file 2. [file elife-71611-supp2.docx]

**Supplementary file 2.** **List of bacterial strains generated in this study.** The sequences of the 5′UTR and N_t_ regions are shown in the Supplementary file 1.

| **Bacterial strain / plasmid** | **Genetic background** | **Source** |
| --- | --- | --- |
| pMR10-BG | *Caulobacter crescentus* NA1000 | This study |
| pMR10-5′UTR*_dnaA_*-N_t_ | *Caulobacter crescentus* NA1000 | This study |
| pMR10-5′UTR*_dnaA_*-ΔN_t_ | *Caulobacter crescentus* NA1000 | This study |
| pMR10-5′UTR*_6/13_*-N_t_ | *Caulobacter crescentus* NA1000 | This study |
| pMR10-5′UTR*_6/13_*-ΔN_t_ | *Caulobacter crescentus* NA1000 | This study |
| pMR10-5′UTR*_lac_*-N_t_ | *Caulobacter crescentus* NA1000 | This study |
| pMR10-5′UTR*_lac_*-ΔN_t_ | *Caulobacter crescentus* NA1000 | This study |
| pMR10-G125C | *Caulobacter crescentus* NA1000 | This study |
| pMR10-C124A | *Caulobacter crescentus* NA1000 | This study |
| pMR10-G126A | *Caulobacter crescentus* NA1000 | This study |
| pMR10-C127A | *Caulobacter crescentus* NA1000 | This study |
| pMR10-U130A | *Caulobacter crescentus* NA1000 | This study |
| pMR10-G132A | *Caulobacter crescentus* NA1000 | This study |
| pMR10-T1 | *Caulobacter crescentus* NA1000 | This study |
| pMR10-T2 | *Caulobacter crescentus* NA1000 | This study |
| pMR10-T3 | *Caulobacter crescentus* NA1000 | This study |
| pMR10-T4 | *Caulobacter crescentus* NA1000 | This study |
| pMR10-T5 | *Caulobacter crescentus* NA1000 | This study |
| pMR10-T6 | *Caulobacter crescentus* NA1000 | This study |
| pMR10-L1 | *Caulobacter crescentus* NA1000 | This study |
| pMR10-L2 | *Caulobacter crescentus* NA1000 | This study |
| pMR10-L3 | *Caulobacter crescentus* NA1000 | This study |
| pMR10-SCM1 | *Caulobacter crescentus* NA1000 | This study |
| pMR10-SCM2 | *Caulobacter crescentus* NA1000 | This study |
| pMR10-ΔP4 | *Caulobacter crescentus* NA1000 | This study |
| pMR10-ΔP5 | *Caulobacter crescentus* NA1000 | This study |
| pMR10-2xN_t_ | *Caulobacter crescentus* NA1000 | This study |
| pMR10-dfsN_t_ | *Caulobacter crescentus* NA1000 | This study |
| pMR10-dfsN_t_ + N_t_ | *Caulobacter crescentus* NA1000 | This study |
| pMR10-mut D1 | *Caulobacter crescentus* NA1000 | This study |
| pMR10-mut D2 | *Caulobacter crescentus* NA1000 | This study |
| pMR10-ΔAAI | *Caulobacter crescentus* NA1000 | This study |
| pMR10-AAI–DDK | *Caulobacter crescentus* NA1000 | This study |
| pMR10-mut CM1 | *Caulobacter crescentus* NA1000 | This study |
| pMR10-mut CM2 | *Caulobacter crescentus* NA1000 | This study |
| pMR10-mut 1 | *Caulobacter crescentus* NA1000 | This study |
| pMR10-mut 2 | *Caulobacter crescentus* NA1000 | This study |
| pMR10-mut 3 | *Caulobacter crescentus* NA1000 | This study |
| pMR10-mut 4 | *Caulobacter crescentus* NA1000 | This study |
| pMR10-mut 5 | *Caulobacter crescentus* NA1000 | This study |
| pMR10-mut 6 | *Caulobacter crescentus* NA1000 | This study |
| pZE12-BG | *Escherichia coli* MG1655 | This study |
| pZE12-5′UTR*_dnaA_*-N_t_ | *Escherichia coli* MG1655 | This study |
| pZE12-5′UTR*_dnaA_*-ΔN_t_ | *Escherichia coli* MG1655 | This study |
| pZE12-5′UTR*_6/13_*-N_t_ | *Escherichia coli* MG1655 | This study |
| pZE12-5′UTR*_6/13_*-ΔN_t_ | *Escherichia coli* MG1655 | This study |
| pZE12-dfsN_t_ | *Escherichia coli* MG1655 | This study |
| pZE12-mut D1 | *Escherichia coli* MG1655 | This study |
| pZE12- mut D2 | *Escherichia coli* MG1655 | This study |
| pZE12-ΔAAI | *Escherichia coli* MG1655 | This study |
| pZE12-AAI–DDK | *Escherichia coli* MG1655 | This study |
