# Supplementary material for: A nascent polypeptide sequence modulates DnaA translation elongation in response to nutrient availability
Source: eLife. 2021 Sep 15;10:e71611. doi: 10.7554/eLife.71611 (PMC8443254; doi:10.7554/eLife.71611)
Supplement: Figure 1—figure supplement 2—source data 1. [file h2.pdf › Figure 1-figure supplement 2-source data 1/panel B/CMfinder_results/all/h2.pdf]

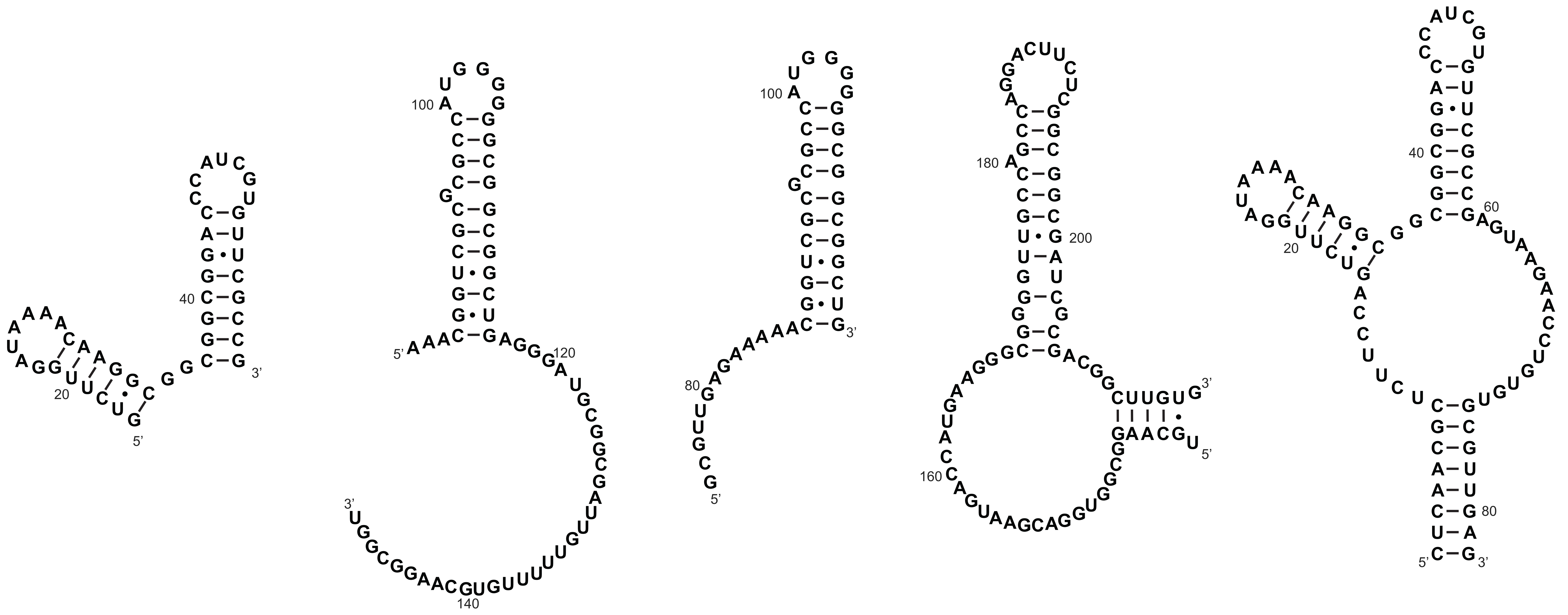
20
40
5'
3'
100
5'
120
3'
140
100
80
3'
5'
180
200
3'
5'
160
40
60
20
80
5'
3'
